# Paediatric invasive group A streptococcal infections and associations with viral infections in 15 European countries after lifting non-pharmaceutical interventions against SARS-CoV-2: an interrupted time-series analysis

**DOI:** 10.1016/j.lanepe.2025.101497

**Published:** 2025-10-18

**Authors:** Léa Lenglart, Izel Özmen, David Aguilera-Alonso, Daniel Blazquez-Gamero, Navin P. Boeddha, Emilie Pauline Buddingh, Danilo Buonsenso, Cristina Calvo, Riccardo Castagnoli, Marta Daina, Maria-Myrto Dourdouna, Marieke Emonts, Carmen Ezinwoke, Catarina Gouveia, David Grandioso Vas, Jeroen Hol, Mette Holm, Christian R. Kahlert, Benno Kohlmaier, Kamila Ludwikowska, Gian Luigi Marseglia, Andrew McArdle, Rachel McGalliard, Athanasios Michos, Anda Nagle, Anita Niederer-Loher, Ulrikka Nygaard, Rianne Oostenbrink, Tina Plankar Srovin, Sandrine Roisin, Jesús Saavedra-Lozano, Adriana Shan, Valtyr Stefansson Thors, Volker Strenger, Angeliki Syngelou, Anne Tilmanne, Monika Tokarczyk, Christo Tsilifis, Maria Tsolia, Adam Tulling, Evelien van Kempen, Mirjam Van Veen, Katarina Vincek, Ulrich Von Both, Dace Zavadska, Mohammed Zaman, Ruud G. Nijman, Naim Ouldali, Dorine Borensztajn, The PEGASUS Consortium, Léa Lenglart, Izel Özmen, David Aguilera-Alonso, Daniel Blazquez-Gamero, Navin P. Boeddha, Emilie Pauline Buddingh, Danilo Buonsenso, Cristina Calvo, Riccardo Castagnoli, Marta Daina, Maria-Myrto Dourdouna, Marieke Emonts, Carmen Ezinwoke, Catarina Gouveia, David Grandioso Vas, Jeroen Hol, Mette Holm, Christian R. Kahlert, Benno Kohlmaier, Kamila Ludwikowska, Gian Luigi Marseglia, Andrew McArdle, Rachel McGalliard, Athanasios Michos, Anda Nagle, Anita Niederer-Loher, Ulrikka Nygaard, Rianne Oostenbrink, Tina Plankar Srovin, Sandrine Roisin, Jesús Saavedra-Lozano, Adriana Shan, Valtyr Stefansson Thors, Volker Strenger, Angeliki Syngelou, Anne Tilmanne, Monika Tokarczyk, Christo Tsilifis, Maria Tsolia, Adam Tulling, Evelien van Kempen, Mirjam Van Veen, Katarina Vincek, Ulrich Von Both, Dace Zavadska, Mohammed Zaman, Ruud G. Nijman, Naim Ouldali, Dorine Borensztajn, Floor Dekkers, Christian Giske, Rachel Hawkins, Olof Hertting, Mojca Kolnik, Enitan Carrol, Nadia Lewis-Burke, Marko Pokorn, Pratham Raghoenath, Samuel Rhedin, Wouter Rozemeijer, Katja Seme, Natalia Syrimi, Elizabeth-Barbara Tatsi, Ivana Velimirovic, Selina Wilfing, Holger Till, Mario Ramirez, Ana Friães

**Affiliations:** a Hôpital Universitaire Robert Debré, Paediatric Emergency Department, 48 Boulevard Sérurier, 75019, Paris, France; b EPIC Team, IAME Laboratory, UMR1137, University Paris Cité, Paris, France; c Northwest Clinics, Department of Paediatrics, Wilhelminalaan, 12, 1815 JD, Alkmaar and Den Helder, Netherlands; d Erasmus Medical Centre Sophia Children's Hospital, Department of General Paediatrics, Dr. Molewaterplein 40, 3015 GD, Rotterdam, Netherlands; e Gregorio Marañón Hospital, Department of Paediatrics, Instituto de Investigación Sanitaria Gregorio Marañón (IiSGM)Universidad Complutense, Madrid, Spain; f Hospital Universitario 12 de Octubre, Paediatric Infectious Diseases Unit, Instituto de Investigación Hospital 12 de Octubre (i+12), Universidad Complutense, CIBERINFEC, Av. de Córdoba, s/n, 28041, Madrid, Spain; g Department of Paediatrics, Maasstad Hospital, Maasstadweg 21, 3079 DZ, Rotterdam, Netherlands; h Willem-Alexander Children's Hospital, Leiden University Medical Centre, Albinusdreef 2, 2333 ZA, Leiden, Netherlands; i Area Paediatrica, Dipartimento di Scienze Della Vita e Sanità Pubblica, Università Cattolica Del Sacro Cuore, Roma, Italy; j Department of Woman and Child Health and Public Health, Fondazione Policlinico Universitario A. Gemelli IRCCS, Rome, Italy; k Department of Paediatric Infectious Diseases, Hospital Universitario La Paz, Fundación IdiPaz, CIBERINFEC, P° Castellana, 261, 28046, Madrid, Spain; l Paediatric Unit, Department of Clinical, Surgical, Diagnostic and Paediatric Sciences, University of Pavia, Pavia, Italy; m Fondazione IRCCS Policlinico San Matteo, Paediatric Clinic, Piazzale Golgi, 19, 27100, Pavia, Italy; n Department of Paediatrics, Riga Stradins University, Vienības Gatve 45, LV1004, Riga, Latvia; o First Department of Paediatrics, Aghia Sophia Children's Hospital, National and Kapodistrian University of Athens, Thivon and Levadeias, 11526, Athens, Greece; p Newcastle Upon Tyne NHS Hospitals Foundation Trust, Great North Children's Hospital, Paediatric Immunology, Infectious Diseases & Allergy, Newcastle, United Kingdom; q Newcastle University, Translational and Clinical Research Institute, Queen Victoria Road, RVI CRB Level 4 Block 2, NE1 4LP, Newcastle upon Tyne, Newcastle, United Kingdom; r Infectology Unit, Department of Paediatrics, Hospital Dona Estefania, Rua Jacinta Marto, 1169-045, Lisbon, Portugal; s Aarhus University Hospital, Department of Paediatrics and Adolescent Medicine, Palle Juul-Jensens Boulevard 99, 8200, Aarhus, Denmark; t Division of Paediatric Infectious Diseases and Infection Control, Children's Hospital of Eastern Switzerland, St. Gallen, Claudiusstrasse 6, 9006, St. Gallen, Switzerland; u Division of General Paediatrics, Medical University of Graz, Auenbruggerplatz 34/2, 8036, Graz, Austria; v Department of Paediatric Infectious Diseases, Wroclaw Medical University, Chalubinskiego 2-2a, 50-368, Wroclaw, Poland; w Department of Infectious Diseases, Alder Hey Children's Hospital, East Prescot Road, L12 2AP, Liverpool, United Kingdom; x Department of Paediatrics and Adolescent Medicine, Copenhagen University Hospital, Rigshospitalet, Blegdamsvej 9, 2100, Copenhagen, Denmark; y Department of Infectious Diseases, University Medical Centre Ljubljana, Japljeva 2, 1241, Ljubljana, Slovenia; z Infectious Diseases Department, CHU-Tivoli, Avenue Max Buset, 34, 7100, La Louvière, Belgium; aa Department of Infectious Diseases, Children's Hospital Iceland, Landspitali University Hospital Iceland, Hringbraut, 101, Reykjavik, Iceland; ab Second Department of Paediatrics, National and Kapodistrian University of Athens (NKUA), School of Medicine, P. and A. Kyriakou Children's Hospital, Thivon and Levadias St, 11527, Athens, Greece; ac Department of Paediatrics, Juliana Children's Hospital, Haga Hospital, Els Borst Eilersplein 275, 2545 AA, The Hague, Netherlands; ad Division of Paediatric Infectious Diseases, Dr. von Hauner Children's Hospital, University Hospital, Ludwig-Maximilians-University, Munich, Germany; ae Department of Paediatric Emergency Medicine, St Mary's Hospital - Imperial College NHS Healthcare Trust, Praed Street, W2 1NY, London, United Kingdom; af Centre for Paediatrics and Child Health, Section of Paediatric Infectious Diseases, Department of Infectious Diseases, Imperial College London, Faculty of Medicine, Imperial College, London, United Kingdom; ag Department of General Paediatrics and Paediatric Infectious Diseases, Hôpital Universitaire Robert Debré, 48 Boulevard Sérurier, 75019, Paris, France

**Keywords:** Streptococcus pyogenes, Group A streptococcus, Invasive streptococcal disease, Outbreak, Paediatrics, Viral infections, Viruses, Influenza, Flu, RSV, Respiratory syncytial virus, VZV, Varicella, Immunity debt, Non pharmaceutical interventions, Europe

## Abstract

**Background:**

After lifting non-pharmaceutical interventions (NPIs) against the transmission of SARS-CoV-2, various countries experienced an increase in invasive Group A Streptococcal (iGAS) infections. We aimed to characterise the paediatric outbreak across Europe and to analyse the influence of viral infections.

**Methods:**

We conducted an interrupted time-series analysis based on data from 15 European countries from the PEGASUS consortium. We assessed the evolution of the number of iGAS cases aged 1 month to 18 years between 01/01/2018 and 03/31/2024, comparing the post-NPIs period (01-04-2022 until 31-03-2024) to the baseline period (01-01-2018 until 31-03-2020). Further analyses were performed by country, clinical phenotype, age and severity, including sensitivity analyses. We then explored whether certain iGAS phenotypes correlated with trends in RSV, influenza and VZV across countries over time using Google Trends data.

**Findings:**

We included 2091 iGAS cases over the study period; 79 children (3.6%) died and 580 (27.7%) required PICU admission. We estimated an overall increase of +229.8% (95% CI (141.9–341.6)) among iGAS cases from October 2022 to March 2024, compared to the baseline period. The observed increases varied across clinical phenotypes, ranging from +62.7% (95% CI (8.3–157.9)) for osteo-articular infections to +238.7% (95% CI 75.8–464.8) for pneumonia. We observed a strong correlation between the incidence of iGAS pneumonia and RSV (Rho: 0.57, 95% CI [0.11–0.79]) and influenza (Rho 0.69, 95% CI 0.35–0.87); and between skin and soft tissue infections and VZV (Rho: 0.73, 95% CI [0.42–0.89]).

**Interpretation:**

The patterns observed across Europe during this outbreak demonstrate an association between respiratory viruses as well as VZV, and iGAS.

**Funding:**

This study has received funding from 10.13039/100013543ESPID, INOPSU and the Northwest Clinics. The COPP study group was supported by grants of the Dutch National Health Council (ZonMW) project number 10430072110007 and the Christine Bader Foundation.

**Research in context**

**Evidence before this study**

At the end of 2022, several public health agencies raised alerts regarding an unusual increase in invasive group A streptococcal (iGAS) infections among children. We searched PubMed for studies investigating clinical manifestations and epidemiology of iGAS infections in children aged 0–18 years, published before April 30, 2025, on outbreaks between 2018 and 2024, written in English. We used the search terms: “invasive group A streptococcus” AND “paediatric” AND “Outbreak” and synonyms of the previously mentioned terms. Several studies described increased paediatric iGAS incidence and explored potential drivers, such as the emergence of more virulent emm-types and reduced population immunity following periods of non-pharmaceutical interventions (NPIs) during the COVID-19 pandemic. The potential role of viral co-infections in predisposing to iGAS has been highlighted in both recent and pre-pandemic studies. However, most prior reports were based on single-country data.

**Added value of this study**

This time series analysis compared the dynamics of different iGAS clinical phenotypes between European countries in the recent outbreak and explored the potential influence of seasonal viruses in driving this outbreak. To our knowledge, this is the largest European study population investigating iGAS disease.

Our findings demonstrate heterogeneity in iGAS clinical presentations across countries and suggest that increases in iGAS pneumonia were temporally associated with RSV and influenza activity, while skin and soft tissue iGAS infections tracked with VZV trends. This multi-country approach strengthens the evidence for viral co-circulation as a potential trigger for iGAS and provides insight into regional variation in disease burden and presentation.

**Implications of all the available evidence**

These findings support existing hypotheses that disruptions in viral transmission dynamics—such as those caused by NPIs—may influence iGAS epidemiology. Future pandemic preparedness should account for how changes in the circulation of common viral pathogens could influence the incidence of secondary bacterial infections, especially among vulnerable paediatric populations. Targeted immunisation strategies against viruses such as RSV, influenza, and VZV could play a role in reducing the burden of iGAS and should be considered as part of broader infectious disease prevention frameworks.

## Introduction

Group A Streptococcal disease (iGAS) is estimated to cause 198.000 deaths worldwide,^1^ mostly among children and the elderly. Group A Streptococcus (GAS), commonly referred to as *Streptococcus pyogenes*, is associated with a broad clinical spectrum, ranging from asymptomatic carriage to invasive disease, with presentations including septic arthritis, streptococcal toxic shock syndrome, and meningitis. The ability for GAS to invade depends on host, bacterial and environmental factors. As an example, viral co-infections^2^^,^^3^ have been previously associated with iGAS.

During the COVID-19 pandemic, non-pharmaceutical interventions (NPIs) were implemented worldwide to reduce the spread of SARS-CoV-2 with major impacts on the frequency of other viral or bacterial infections in children and adults.^4^, ^5^, ^6^, ^7^ Although a decrease in infections was observed during the implementation of NPIs,^8^ after lifting NPIs, important outbreaks of respiratory viruses^9^^,^^10^ (Respiratory Syncytial Virus (RSV), Influenza) and invasive bacterial infections^11^ were reported in many countries.

The end of 2022 was marked by alerts from several public health agencies^12^ regarding unusually high numbers of iGAS infections in the paediatric population. Since then, several studies have described this iGAS outbreak.^13^, ^14^, ^15^ However, the underlying mechanisms driving this outbreak remain incompletely understood.

The aim of this study was to characterise these iGAS outbreaks at a large-scale multinational level and to explore the potential role of the unprecedented rise in viral infections in driving this outbreak across Europe in the context of lifting NPIs.

## Methods

### Setting, study design and inclusion criteria

We conducted an interrupted time-series analysis based on data from a multinational observational cohort study of paediatric iGAS infections, the PEGASUS consortium.

We included all patients aged 1 month to 18 years old, admitted to one of the participating centres and diagnosed with an iGAS infection.

An iGAS infection was defined as 1) a clinical presentation consistent with iGAS (such as sepsis, septic shock, streptococcal toxic shock syndrome (defined according to CDC 2010),^16^ meningitis, pneumonia, osteoarticular infection, myositis, necrotising fasciitis) and the isolation of GAS by culture, molecular detection by PCR or antigen detection test from a normally sterile body site; or 2) a clinical presentation consistent with necrotising fasciitis or streptococcal toxic shock syndrome with isolation of GAS from a non-sterile body site, with no evidence for another pathogen explaining the clinical presentation. For this study, clinical phenotypes were not exclusive, and patients could be diagnosed with one or more clinical phenotypes.

### The PEGASUS consortium

The participating centres were all part of the Paediatric European Group A Streptococcus United Study (PEGASUS) consortium, a research network set up in 2023 to investigate iGAS cases in children across Europe. The consortium consists of tertiary/academic, teaching as well as non-teaching/district general hospitals across 17 European countries, of which 15 provided data for this specific study (See Appendix 1). The consortium aims to aid in early recognition and to describe the incidence, risk factors, clinical phenotypes, microbiology and resistance, treatment and outcomes for iGAS in children across Europe.

Individual sites have entered data into the secured PEGASUS database. For these individual study sites, data were collected retrospectively from January 2018 to April 2023, and prospectively from April 2023 onwards, with prospective data collection still ongoing. Data on baseline characteristics, symptoms and vital functions at first presentation, diagnosis, treatment and outcome (death, PICU admission, long term sequelae, days of hospitalisation) were collected. In addition to the individual study sites, several existing European registries—both regional and national, and either retrospective or prospective in nature—that had initially collected data independently, have joined the PEGASUS consortium. Data from these registries were then combined with the PEGASUS database after joining the consortium (see Appendix 1). Several aspects of the data collection—such as antibiotic and IVIG treatment, differences in clinical practices between countries (e.g., post-exposure prophylaxis), and variation in emm types—will be addressed in future papers.

### Study periods

For this study, the study period spans from January 1, 2018, to March 31, 2024. The intervention was defined as the implementation of NPIs against SARS-CoV-2. To define the dates of the different study periods, we used the *stringency index* developed by Our World In Data^17^ during the pandemic. This index is a composite measure based on nine response indicators including school closures, workplace closures and travel bans, rescaled to a value from 0 to 100 (100 = strictest). By March 2022, most of the participating countries (12/15) had a low stringency index (<40% for the total population weighted according to SARS-CoV-2 vaccination status rules).

This allowed us to define three periods for the further analyses (See Appendix 2): the *baseline period*, the *NPIs period* and the *post-NPI period*. The *baseline period* spans from January 1st, 2018, to March 31st, 2020. The *NPIs period* spans from April 1st, 2020, to March 31st^,^ 2022. The *post-NPIs period* spans from April 1st, 2022, to March 31st, 2024.

### Data on respiratory viruses and VZV

For each participating country, data on the evolution of respiratory viruses (RSV and influenza) and VZV epidemics were obtained using Google Trends data searches from January 1st, 2018, to March 31st, 2024. Google Trends data are given by month and represent the relative search interest for a specified topic in a given country and time period: the month assigned a value of 100 is the one with the highest search volume during the study period; the other months are expressed as a percentage relative to this maximum.^18^ To study the epidemiological trend of infectious diseases, Google Trends data have shown to be reliable proxies of laboratory-confirmed data or Emergency Department (ED) visits; particularly in terms of temporal accuracy as well as relative magnitude of viral circulation.^19^, ^20^, ^21^, ^22^, ^23^ This means they can be used to estimate the timing and relative intensity of viral epidemics. Moreover, Google Trends provides previously validated *topics* rather than raw search terms. In our case, we used the topic “bronchiolitis” as a proxy for RSV^23^; “flu” for influenza^24^ and “chickenpox” for VZV.^25^ *Topics* are considered more reliable than keywords, as they capture various spelling variants, acronyms, and translations across languages-which is particularly relevant in a multi-country European study. (See Appendix 3). To account for differences in national search behaviour, we first extracted country-specific Google Trends data and calculated the average monthly search index across countries, resulting in a composite European trend for each viral infection. We then correlated this composite indicator with the monthly crude number of iGAS cases (e.g., pneumonia or SSTI), aggregated across the same countries. To assess the validity of using Google Trends data in the context of our study, we obtained syndromic and microbiological surveillance data for RSV, influenza, and VZV from a subset of participating countries^26^, ^27^, ^28^, ^29^, ^30^ and assessed the correlation between national surveillance data and search volumes at a national level.

### Outcome measure

The main outcome was the monthly number of iGAS infections over time assessed by interrupted time-series analysis models at a multinational level.

Secondary outcomes included the evolution of number of iGAS cases in subgroups: (i) at national levels in countries including more than 50 cases, (ii) by age-group (under 2 years-old, between 2 and 5 years-old, older than 5 years-old), (iii) by clinical phenotype (categorised as: bacteraemia or sepsis including streptococcal toxic shock syndrome (STSS), ear-nose-throat (ENT) infection or abscess, skin and soft tissue infections (SSTI) including necrotising fasciitis, pneumonia and pleural empyema and osteoarticular infection, see Appendix 4) and (iv) by severity of each case (general paediatric ward hospitalisation or paediatric intensive care unit (PICU) hospitalisation).

### Statistical analysis

We first described the general characteristics of the patients. To assess whether the impact of the interventions differed by age group, country, clinical phenotype, or disease severity, we performed a Type II analysis of deviance (ANOVA). Subsequently, we conducted interrupted time-series analyses to evaluate the evolution of iGAS infection numbers in Europe following the lifting of NPIs. Interrupted time series analyses allow to estimate the fitted value of the number of observed iGAS infections after the lifting of NPIs, compared to a counterfactual scenario in which no NPIs were implemented, based on the baseline period parameters. The outcome was analysed using a multilevel negative binomial regression model taking into account the heterogeneity across countries and accounting for seasonality, secular trend and overdispersion of data.^31^^,^^32^ The time unit of one month was chosen. Monthly case counts were aggregated by country. Time variables and seasonal harmonics were included as covariates. Two intervention indicators and their post-intervention time trends were modelled to capture changes associated with NPIs implementation and lifting. The model incorporated a random intercept for countries to account for clustering. The validity of the multilevel regression model was assessed by visual inspection of correlograms and residual analysis (See Appendix 5).

We assumed that the introduction of NPIs had an immediate effect, while their lifting would not lead to an immediate rebound, but rather a delayed response. To reflect this assumption in the model, we introduced a 6-month lag period (April 1st–September 30th, 2022) following lifting NPIs, during which no immediate change was expected. This period was included in the time series but without modelling a change in level or slope, to account for a plausible delayed effect. The overall increase in cases during the post-NPIs period was then assessed from October 1st, 2022, to March 31st, 2024. Confidence intervals were estimated via parametric bootstrap by simulating model coefficients from their estimated covariance matrix, recalculating the predicted increases over multiple iterations.

A range of sensitivity analyses were performed to assess the robustness of the findings of the model. We performed different types of regressions to test the overdispersion of data: (i) using a 3-segment linear segmented regression; (ii) using a single level binomial negative model, (iii) using a quasi-Poisson regression model accounting for 9- and 12- month seasonality. Finally, we did not include the 6-month lag period and calculated the overall increase during the entire post-NPIs period using the main multilevel negative binomial regression.

The main multilevel model was also used for secondary analysis based on multi-country data (by age-group; clinical phenotype and severity of the cases). However, to investigate country-specific effects and address overdispersion more directly within each context, separate quasi-Poisson models were fitted for each country.

Then, to explore the relationship between the various clinical phenotypes of iGAS and viral dynamics, we performed Pearson correlation tests between the trends of RSV, influenza and VZV and the overall monthly number of specific iGAS phenotypes of iGAS (pulmonary and SSTI cases). Moreover, exploratory analyses were performed on the other clinical phenotypes included in this study.

All centres (except Iceand, 17 cases) participated in all time points. Analyses were performed on complete datasets only. All statistical tests were two-sided, with p < 0.05 considered statistically significant. All analyses were performed using R statistical software, version 4.1.1 (http://www.R-project.org).

### Ethics

The study protocol was approved by the Medical Research Ethics Committees United (MEC-U) in the Netherlands and the local institutional review board of each participating centre. As this was an observational study, the participants did not undergo an intervention for research purposes, and exclusively anonymous data were collected in a secured database, this study was exempt from informed consent by the local institutional review board of most participating centres. In some of the participating centres, the local institutional review board required informed consent, which was then obtained for all patients (See Appendix 1). Some national sites collected pseudonymous data from prospectively included participants with informed consent, but limited, aggregated and anonymous data was used for the current study).

### Role of the funding source

The funders of the study did not have any role in the design of the study, data collection, data analysis, interpretation of the results or writing of the manuscript.

## Results

### General characteristics of the population

We included a total of 2091 paediatric iGAS cases over the study period among 15 European countries. Children aged under two years accounted for 22.3% (466/2003) of the study cases, while those between 2 and 5 years accounted for 21.5% (449/2091) and 34.2% (716/2091) for those over 5 years. Among the cases, 580/2091 (27.7%) required PICU admission and 79/2091 (3.7%) deaths at 30 days were reported (See Table 1).

**Table 1:** Characteristics of iGAS cases across 15 European countries from January 1st 2018 to March 31st 2024, N = 2091.

	NUMBER OF CASES, N (%)				
	Total	Baseline period^a^	NPIs period^a^	Post-NPIs period^a^	ANOVA^b^
All iGAS cases	2091	511	136	1444	–
Cases aged under 2	466 (22.3)	141 (27.6)	26 (19.1)	299 (20.7)	Χ^2^ = 2.9 p-value = 0.02
Cases aged 2–5	449 (21.5)	108 (21.1)	36 (26.5)	305 (21.1)	
Cases older than 5	716 (34.2)	172 (33.7)	29 (21.3)	515 (35.7)	
*Missing data for age*	*460 (22.0)*	*90 (17.6)*	*45 (33.1)*	*325 (22.5)*	
Austria	22 (1.1)	7 (1.4)	3 (2.2)	12 (0.8)	Χ^2^ = 17.8 p-value <0.001
Belgium	6 (0.3)	1 (0.2)	2 (1.5)	3 (0.2)	
Denmark	128 (6.1)	43 (8.4)	7 (5.1)	78 (5.4)	
France	72 (3.4)	18 (3.5)	2 (1.4)	52 (3.6)	
Greece	42 (2.0)	7 (1.4)	1 (1.0)	34 (2.4)	
Iceland	17 (0.8)	0 (0)	0 (0.0)	17 (1.2)	
Italy	12 (0.6)	3 (0.6)	0 (0.0)	9 (0.6)	
Latvia	198 (9.5)	59	11 (8.1)	128 (8.9)	
Netherlands	541 (25.9)	108	51 (37.5)	382 (26.5)	
Poland	8 (0.4)	3 (0.6)	0 (0.0)	5 (0.3)	
Portugal	179 (8.6)	49 (11.5)	4 (2.9)	126 (8.7)	
Spain	129 (6.2)	42 (8.6)	14 (10.3)	73 (5.1)	
Slovenia	46 (2.2)	12 (2.5)	3 (2.2)	31 (2.1)	
Switzerland	531 (25.4)	106 (20.7)	24 (17.6)	401 (27.8)	
United Kingdom	160 (7.7)	53 (10.4)	14 (10.3)	93 (6.4)	
Bacteriemia and sepsis cases (including STSS)	797 (38.1)	211 (41.3)	53 (39.0)	533 (36.9)	Χ^2^ = 24.8 p-value <0.001
STSS cases	93 (4.4)	20 (3.9)	8 (5.9)	65 (4.5)	
ENT and abscess cases	534 (25.5)	129 (25.2)	27 (19.9)	378 (26.2)	
SSTI (including necrotizing fasciitis)	443 (21.2)	100 (19.6)	35 (25.7)	308 (21.3)	
Necrotizing fasciitis cases	87 (4.2)	16 (3.1)	3 (2.2)	68 (4.7)	
Pneumonia cases	473 (22.6)	108 (21.1)	21 (15.4)	344 (23.8)	
Osteo-arthritis cases	337 (16.1)	90 (17.6)	26 (19.1)	221 (15.3)	
Meningitis cases	81 (3.9)	20 (3.9)	2 (1.5)	59 (4.1)	
General ward admission	1483 (70.9)	375 (73.4)	105 (77.2)	1003 (69.5)	Χ^2^ = 3.2 p-value = 0.07
Intensive care unit admission	580 (27.7)	124 (24.3)	31 (22.8)	425 (29.4)	
*Missing data for admission type*	*28 (1.3)*				
Death	79 (3.8)	17 (3.3)	8 (5.9)	54 (3.7)	–

Abbreviations: NPIs, non-pharmaceutical interventions; iGAS, invasive Group A streptococcus; ENT, Ear–Nose-Throat; STSS, Streptococcal Toxic Shock Syndrome; SSTI, Skin and soft tissue infection.

a For the study, we defined three study periods: the baseline period: from January 1st, 2018, to March 31st, 2020, the NPIs period from April 1st, 2020, to March 31st, 2022, and the post-NPIs period: from October 1st, 2022, to March 31st, 2024.

b To test whether the effect of the intervention differed by age groups, countries, clinical phenotypes, or severity, we performed a Type II analysis of deviance (ANOVA).

Overall, the distribution of cases across age categories, participating countries, and clinical phenotypes varied significantly over the three time periods (see Table 1). Missing values were less than 0.5% for all variables, except for age, which was only available in age categories for patients from the Dutch Consortium and ENT or abscesses which was not available for the Danish cohort.

### Epidemiological evolution of iGAS during the post-NPIs period

**Fig. 1:**
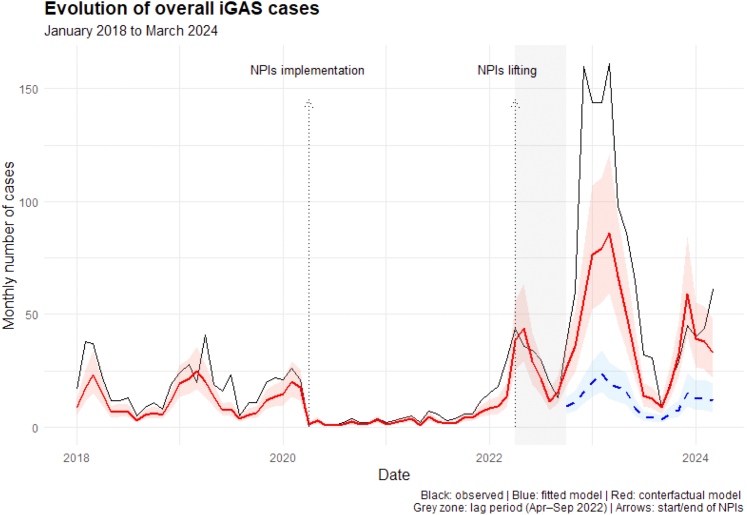
**Evolution of the monthly number of iGAS cases from January 1st, 2018, to March 31st, 2024, assessed by interrupted time series analyses, N = 2091**. For the study, we defined three study periods: the baseline period: from January 1st, 2018, to March 31st, 2020, the NPIs period from April 1st, 2020, to March 31st, 2022, and the post-NPIs period: from October 1st, 2022, to March 31st, 2024. The effect of the implementation of NPIs was considered immediate, while the effect of their lifting was considered delayed and not expected to be immediate. Thus, we defined a lag period of 6 months from April 1st to September 30th 2022, highlighted in grey. The black line corresponds to the observed monthly number of iGAS cases. The red line corresponds to the fitted value of the monthly number of cases with its 95% confidence interval. The dashed blue line and its 95% confidence interval corresponds to the expected number of iGAS cases during the post-NPIs period, if no NPIs had been implemented. Abbreviation: NPIs, Non-pharmaceutical interventions; iGAS, invasive Group A streptococcus.

During the study period, we observed an important decrease in iGAS cases during the NPIs period, followed by a strong increase during the post-NPIs period, especially during winter 2022–2023. We estimated a +229.8% (95% CI: [141.9–341.6]) increase in iGAS cases from October 2022 to March 2024 as compared to what would have been expected if no NPIs had been implemented, accounting for previous trends and heterogeneity between countries (See Fig. 1). This finding was supported by sensitivity analyses (See Table 2).

**Table 2:** Overall increase in the monthly number of iGAS cases during the post-NPIs period, associated to sensitivity analyses and subgroup analysis by clinical phenotypes of iGAS, N = 2091.

	Overall increase during post-NPIs period^a^	95% CI
All iGAS cases	+229.8%	141.9–341.6
Sensitivity analysis n°1: linear segmented regression	+223.9%	89.9–358.0
Sensitivity analysis n°2: single level negative binomial regression	+265.8%	81.1–450.6
Sensitivity analysis n°3: quasi-Poisson regression with 9- and 12- month seasonality	+297.9%	157.1–438.7
Sensitivity analysis n°4: without lag period	+221.1%	137.0–331.5
Bacteriemia and sepsis cases (including STSS)	+177.2%	78.2–299.6
ENT and abscess cases	+177.1%	59.9–351.6
SSTI (including necrotizing fasciitis)	+83.9%	9.5–184.1
Pulmonary cases	+238.0%	75.8–464.8
Osteo-articular cases	+62.7%	−8.3–157.9

The overall increase is calculated as the overall difference between the modulization of the outcome during the post-NPI period and the counterfactual scenario—if no NPIs had been implemented—accounting for the trend of the pre-NPIs period.

Abbreviations: NPIs, non-pharmaceutical interventions; iGAS, invasive Group A streptococcus; ENT, Ear–Nose-Throat; STSS, Streptococcal Toxic Shock Syndrome; SSTI, Skin and soft tissue infection.

a For the study, we defined three study periods: the baseline period: from January 1st, 2018, to March 31st, 2020, the NPIs period from April 1st, 2020, to March 31st, 2022, and the post-NPIs period: from October 1st, 2022, to March 31st, 2024.

The increase varied across age groups, with a stronger estimated increase among older children: +397.2% (95% CI: [188.1–693.3]) compared to younger children (See Appendix 6–8). Moreover, during the post-NPIs period, differences were observed in the increase of iGAS cases across countries: ranging from +159.0% (95% CI: [−32.9 to 351.0]) in the United Kingdom, to +451.7% (95% CI: [228.9–674.4]) in Switzerland (See Appendix 7 and 9).

**Fig. 2:**
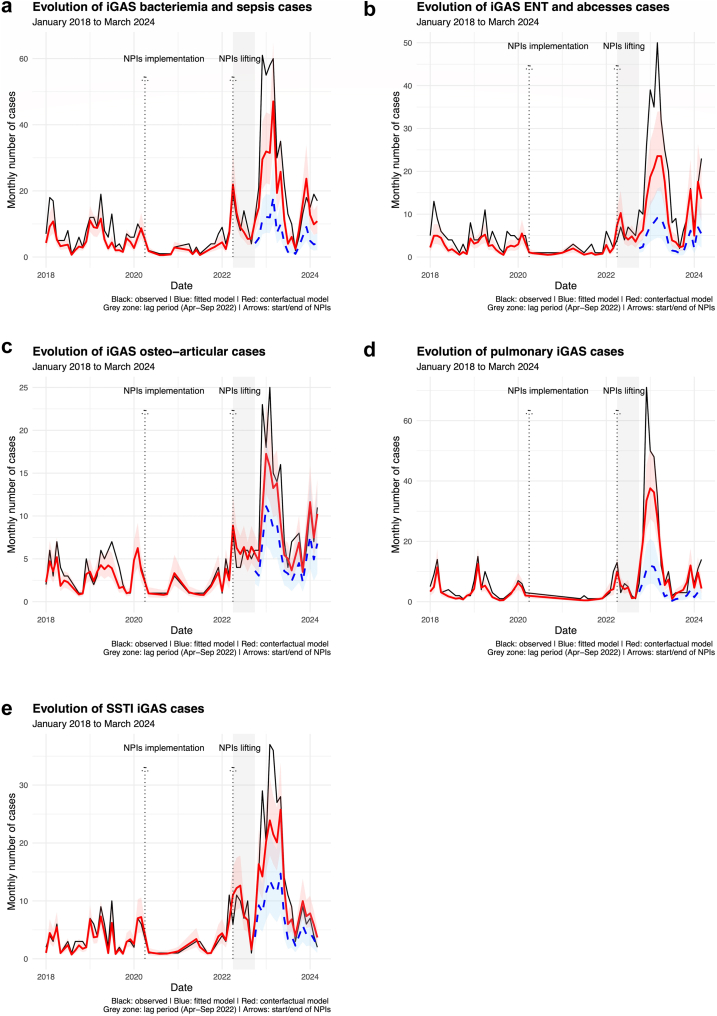
**Evolution of the monthly number of iGAS cases by clinical phenotypes from January 1st 2018 to March 31st 2024 assessed by interrupted time series analyses**. **a**: Bacteriemia and sepsis, including STSS (N = 797); **b**: ENT and abscesses cases (N = 534); **c**: Osteo-articular cases (N = 337); **d**: Pneumonia cases (N = 473); **e**: Cutaneous cases, including necrotizing fasciitis (N = 443). For the study, we defined three study period: baseline period (January 1st, 2018, to March 31st, 2020), NPIs period (April 1st, 2020, to March 31st, 2022), and post-NPIs period (October 1st 2022 to March 31st, 2024.) The effect of the lifting of NPIs was considered delayed, thus, we defined a lag period of 6 months (April 1st to September 30th, 2022). The black line corresponds to the observed monthly number of iGAS cases. The red line corresponds to the fitted value of the monthly number of cases with the 95% confidence interval. The dashed blue line and its 95% confidence interval corresponds to the expected number of iGAS cases during the post-NPIs period, if no NPIs had ever been implemented. Abbreviation: NPIs, Non-pharmaceutical interventions; iGAS, invasive Group A streptococcus; ENT, Ear-Nose-throat; STSS, streptococcal toxic shock syndrome; SSTI, Skin and soft tissue infections.

Among the clinical phenotypes, the increase in iGAS cases ranged from +62.7% (95% CI: [−8.3 to 157.9]) for osteoarticular cases to +238.0% (95% CI: [75.8–464.8]) for pulmonary cases (See Table 2 and Fig. 2). Furthermore, the clinical phenotype that showed the greatest increase varied between countries (See Appendix 10). For example, the increase primarily involved pulmonary cases in the Netherlands and the United Kingdom, whereas in countries such as Denmark, Latvia, and Switzerland, it mainly involved SSTI cases. Paediatric ED visits and hospital admissions in 2023 for each hospital are shown in Appendix 11. Finally, similar ranges of increases were observed among the different levels of severity of the iGAS cases (See Appendix 7 and 8).

### Correlation between the evolution of respiratory virus, VZV and clinical phenotypes of iGAS

**Fig. 3:**
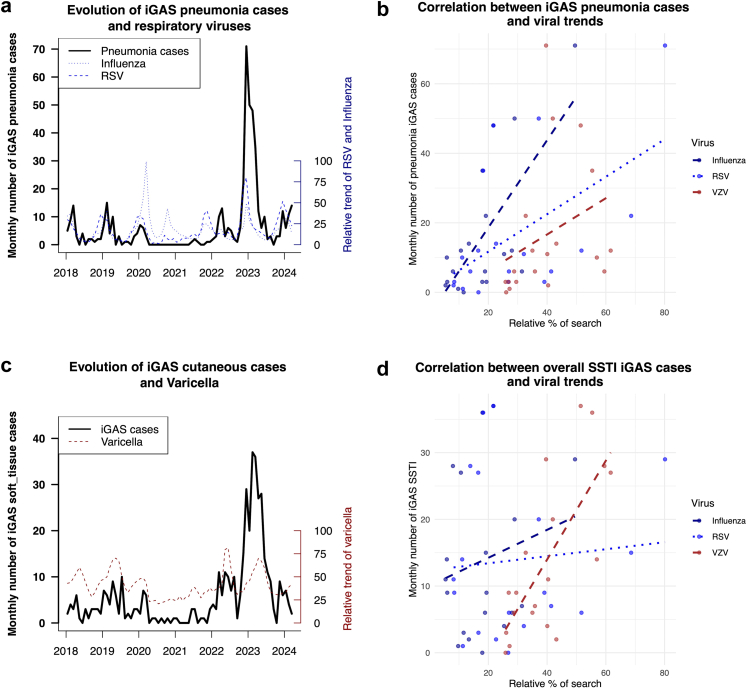
**Correlation between the evolution of viruses (Influenza, RSV and varicella) and clinical phenotypes of iGAS**. **a**: Evolution of iGAS pneumonia cases (N = 473), Influenza and RSV from January 1st, 2018, to March 31st, 2024. **b**: Graphical representation of the Pearson correlation between the monthly number of iGAS pneumonia cases and the relative trend of viruses (Influenza, RSV and VZV) during the post NPIs period. **c**: Evolution of SSTI iGAS cases (N = 443) and VZV from January 1st, 2018, to March 31st, 2024. **d**: Graphical representation of the Pearson correlation between the monthly number of SSTI iGAS cases and the relative trend of viruses (Influenza, RSV and VZV) during the post NPIs period. Abbreviations: NPIs, non-pharmaceutical interventions; iGAS, invasive Group A streptococcus; SSTI, Skin and soft tissue infection.

We observed a strong to very strong correlation between the evolution of Google Trends search data for “bronchiolitis,” “flu,” and “varicella” and the syndromic or microbiological national surveillance data for RSV, influenza, and varicella in a subgroup of countries. The Pearson's correlation coefficients between Google Trends and National surveillance data ranged from 0.66 (CI 0.51–0.77) to 0.95 (CI 0.92–0.97) for varicella, from 0.38 (CI 0.09–0.61) to 0.69 (CI 0.49–0.82) for influenza (laboratory confirmed, syndromic or both) and from 0.34 (CI 0.12–0.52) to 0.73 (CI 0.58–0.83) for bronchiolitis (laboratory confirmed and syndromic) (Appendix 12–15). The correlation to respiratory viruses and VZV varied across clinical phenotypes. A strong Pearson correlation was observed between the evolution of VZV and the number of iGAS SSTI cases (Rho: 0.73, 95% CI [0.42–0.89]) during the post-NPIs period, whereas the Pearson correlation was lower and non-significant between respiratory viruses and SSTI cases. On the other hand, we observed a strong Pearson correlation between respiratory viruses and the number of pneumonia iGAS cases during the post-NPIs period (influenza Rho: 0.69 95% CI [0.42–0.89] and RSV Rho: 0.57; 95% CI [0.11–0.79]) and a lower and non-significant Pearson correlation to VZV infections (See Table 3 and Fig. 3). See Appendix 16 for exploratory analyses examining the associations between respiratory viruses, VZV and the other clinical phenotypes included in the study.

**Table 3:** Pearson correlation tests between virus (Influenza, RSV and varicella) epidemiological trend and SSTI (N = 443) or pneumonia (N = 473) cases during the post-NPIs period.

Correlation between	SSTI iGAS cases			Pneumonia iGAS cases		
	Rho	95% CI	p-value	Rho	95% CI	p-value
Influenza	0.19	−0.28–0.59	0.43	0.69	0.35–0.87	**<0.001**
RSV	0.08	−0.38–0.52	0.72	0.57	0.11–0.79	**0.01**
VZV	0.73	0.42–0.89	**<0.001**	0.31	−0.16–0.67	0.19

Abbreviations: NPIs, non-pharmaceutical interventions; iGAS, invasive Group A streptococcus; SSTI, Skin and soft tissue infection.

Bold entities indicate p < 0.05.

## Discussion

This multinational study of the 2022–2023 iGAS outbreak estimated an overall increase of +297% of iGAS cases during the post-NPIs period compared to the pre-pandemic period and unveiled important variation of clinical phenotypes across European countries. These variations seem to be related to differences in the evolution of VZV and respiratory viruses (RSV and influenza) cases across European countries during the post-NPIs period, highlighting the relationship between viral dynamics and iGAS evolution.

Our findings are in line with several other studies describing an increase in paediatric iGAS.^13^, ^14^, ^15^ In addition to an increase in paediatric iGAS cases, several studies have described a similar increase in adult cases of iGAS as well. Studies comparing adults and children showed that respiratory infections were more frequently observed in paediatric patients, whereas non-respiratory infections, such as SSTI, were more common in adults.^33^ Preceding viral infections were reported more often in children than in adults.^3^ Most children with invasive GAS disease had been previously healthy with no underlying conditions, while the majority of adults had chronic comorbidities. Although ICU admission rates were higher in children, mortality was higher among adults.^33^

The recent surge in paediatric invasive group A streptococcal (iGAS) infections likely results from a combination of environmental, host and pathogen factors– and their interaction. First, our findings are consistent with the hypothesis that immune debt played a contributory role in the outbreak^34^ secondary to the implementation of NPIs. Reduced exposure to common respiratory viruses as well as to GAS during the COVID-19 pandemic,^8^ is likely to have diminished population-level immunity. During the post-NPIs period, an increase in GAS carriage was described.^35^^,^^36^ The increase in carriage is likely to have contributed to the increase in invasive GAS, as genetic similarities in strains were found previously.^36^ In line with this, a decrease in non-invasive GAS was observed during the pandemic, followed by an increase in non-invasive GAS in the post-NPIs period. In a previous study, at a country level, the magnitude of the reduction in paediatric respiratory tract infections cases during the implementation of NPIs was associated with the extent of the subsequent increase after their lifting.^37^ Furthermore, a strong correlation was observed between respiratory viruses and pulmonary iGAS cases, with the increase being particularly pronounced among pulmonary cases, which is consistent with previous reports in both children and adults.^3^^,^^33^^,^^38^ Consequently, the strong outbreaks of respiratory viruses, the reduced level of population immunity as well as the increase in GAS carriage^35^ during the post-NPIs period may have provided ideal circumstances for this iGAS outbreak.

Second, viral infections might facilitate bacterial invasion of GAS. Several potential mechanisms have been proposed to explain how VZV might increase the risk of iGAS: the lesions damage the skin barrier and facilitate the invasion of GAS, or VZV infection may lead to a temporary disruption of the immune system, increasing the host's susceptibility to invasive bacterial infections such as iGAS.^39^ The same kind of mechanisms may be involved between respiratory viruses and GAS.^40^ Here, we highlight the epidemiological relationship between, several clinical phenotypes of iGAS and RSV, influenza and VZV. Third, several bacterial factors affecting GAS virulence, including superantigens, toxin profiles and emm types,^41^ may have played a role in the recent surge. Emm types are genetic variations of the emm gene that encodes the M protein in GAS and are used to classify GAS strains.^15^ In a future publication, our study group will examine the emm types in greater detail, as this analysis was beyond the scope of the current study. Together, the interaction between environmental changes, host susceptibility and alterations in pathogen characteristics in the context of lifting NPIs, is likely to have shaped this iGAS outbreak in Europe.

The observed variation in clinical presentations between countries warrants further exploration. Several hypotheses may explain these differences. First, variation in case ascertainment due to differences in hospital admission policies, type of participating centres (e.g., tertiary versus secondary), or clinical thresholds for suspecting and testing for iGAS in specific disease entities, may have introduced selection bias. Second, differences in circulating emm types could play a role, as associations between specific emm types and certain types of infection have been reported. Third, healthcare-seeking behaviour may vary between countries, with some types of infections prompting hospital visits in an earlier phase than others, which then may be managed in outpatient settings. Fourth, community antibiotic prescribing practices could mask or suppress respiratory iGAS presentations prior to hospital admission, particularly if antibiotics are more commonly prescribed for respiratory symptoms. Fifth, differences in viral co-circulation and timing of seasonal epidemics may affect the incidence of specific disease entities associated with iGAS in some regions. Sixth, national immunization policies—such as universal varicella and/or influenza vaccination offered to all children in some countries—may influence the clinical spectrum of iGAS.

### Implications for clinical practice and further research

The correlation between viruses and iGAS identified in this study gives rise to the hypothesis that vaccinating against viral infections, such as influenza, RSV and VZV may represent a potential public health strategy to reduce the burden of iGAS in children. This is especially relevant since a vaccine for GAS is not yet available. However, the potential impact of viral vaccines on iGAS incidence remains uncertain. For instance, although previous studies have shown a decline in varicella-related iGAS following the introduction of VZV vaccination, the overall incidence of iGAS did not decrease.^42^^,^^43^ Furthermore, as RSV immunisation will primarily protect infants in their first year of life, it is unlikely to contribute to herd immunity. Prospective studies are therefore needed to assess whether universal vaccination strategies against these viruses will actually impact the burden of iGAS in children.

Increases in GAS outbreaks following lifting NPIs in the context of COVID-19, and possibly in future pandemics of novel respiratory viruses, highlights the importance of strengthening awareness and timely notification to improve early recognition and management. Further research on virulence factors, such as the M-protein and superantigens, is imperative to enhance comprehension of iGAS pathogenicity and outbreaks.

### Strengths and limitations

To our knowledge this is the largest multinational paediatric consortium investigating iGAS disease. The large study population and multinational design are major strengths of this study. All participating centres used the same definition for iGAS disease, adding to the internal and external validity of our study. Furthermore, ongoing prospective surveillance is a key quality of this study.

The study also has some limitations. First, as this is an observational study, no causal relationship between viral infections and iGAS cases can be established, and similar patterns could be found between other pathogens. However, we performed correlation analyses between the circulation of specific viruses and the clinical phenotypes of iGAS. These analyses revealed a correlation in time and level between these viruses and specific clinical phenotypes of iGAS, suggesting that respiratory viruses and VZV may contribute differently to the epidemiology of specific clinical phenotypes of iGAS. Second, although this consortium comprises a substantial number of countries across Europe, not every European country was represented. Furthermore, the number of participating hospitals per country varied, as in some countries it was a national or regional registry while in other countries individual hospitals participated; however, the total number of countries and patients was large enough to analyse the relationship between viruses and some clinical phenotypes of iGAS. Indications for microbiological testing, in patients presenting with pneumonia for example, cases can differ between countries, which might explain variations in clinical phenotypes per country. Vaccination schedules, especially regarding varicella and influenza vaccination, differ between the participating countries, which probably could have impacted the observed numbers of clinical phenotypes per country, such as pneumonia and SSTI cases. Moreover, we performed interrupted time series analysis taking into account the multi-country aspect of the data. Third, due to our definition of iGAS including only sterile cultures, less severe cutaneous cases, such as cellulitis where the pathogen is often not confirmed, might not have been included. Although we still observed a correlation between SSTI cases and VZV, the actual correlation might be stronger than we identified. Fourth, although our consortium includes four countries with universal VZV immunization programs, assessing the impact of these strategies on SSTI cases was not feasible within our current analysis. Since these vaccination programs were already in place by 2018, there was no change in vaccination policy during the study period, limiting our ability to evaluate their effect using a population-level time series approach. However, as the study continues to recruit prospectively, future analyses will be possible, particularly as the United Kingdom and Slovenia have recently introduced universal VZV vaccination programs. Fifth, having access to individual microbiological or nationally confirmed laboratory data for RSV, influenza, or VZV would have strengthened the findings of this study. However, dedicated national surveillance systems for these viruses exist in only a few of the 15 participating countries. As a result, correlating national surveillance data with the epidemiological trends of various iGAS phenotypes within each country was not feasible. Previous studies have shown that Google Trends data correlate accurately with national surveillance data and ED visits for respiratory infections such as influenza and RSV.^19^, ^20^, ^21^, ^22^, ^23^ To validate our approach using Google Trends data we compared national surveillance data from a subset of countries with their corresponding Google Trends search data and observed mostly strong to very strong correlations. This methodology uniquely enabled us to link the evolution of certain clinical iGAS phenotypes at a European multi-country level to the epidemiological trends of respiratory viruses and VZV. Sixth, the implementation of NPIs varied across countries in both timing and intensity. Although we did not define separate dates for NPI implementation and lifting for each participating country, the multilevel negative binomial regression model, which includes a random intercept for country, allows baseline levels and trends to vary across countries. Seventh, our study did not account for population adherence to NPIs; however, the methodology remained consistent across all countries over time. Eighth, healthcare seeking behaviour and access to medical help may have differed throughout the study period due to the pandemic and therefore delays in diagnosis or hospital presentation could have occurred. However, the estimates of the increase in cases requiring ICU did not statistically differ from cases admitted to wards. Ninth, the use of composite categories for several clinical phenotypes may have introduced some heterogeneity, however, efforts were made to harmonize definitions across countries through broader composite categories to allow for meaningful aggregation without compromising core clinical distinctions.

In conclusion, taking advantage of a large multinational surveillance system of iGAS, the PEGASUS consortium, this study highlighted the differences in magnitude and clinical phenotypes involved in the 2022–23 iGAS outbreak across countries in Europe, supporting the role of viruses as potential drivers of this outbreak.

## Contributors

All authors contributed substantially to the conception and design of the work, as well as to the acquisition and interpretation of data. All authors contributed to the critical revision of the manuscript for important intellectual content, and all approved the final version of the manuscript. All authors agree to be accountable for all aspects of the work.

All authors of the PEGASUS consortium contributed substantially to the conception and design of the work, and to the interpretation of data. Consortium authors contributed to the critical revision of the manuscript and approved the final version of the manuscript, and agree to be accountable for all aspects of the work.

LL, DB, IO and NO verified and analysed the data. LL, DB, NO and IO interpreted the data and wrote the first draft for the manuscript. The corresponding author affirms that all listed authors meet the authorship criteria and that no authors have been omitted.

## Data sharing statement

Deidentified data will be made available to researchers upon reasonable request, subject to the submission of a methodologically sound research proposal. Data will be shared only to the extent necessary to answer the proposed research question and only if there is no overlap with ongoing analyses within the PEGASUS consortium.

## Declaration of interests

DBG and EvK declare receiving a honorarium from MSD for a presentation. RO declares receiving fees from EUSEM for attending a medical conference. AS declares receiving fees from Angelini Pharma for attending medical conferences and scientific meetings. VT declares receiving consulting fees from Sanofi. MT declares receiving fees from MSD and Pfizer for attending medical conferences and scientific meetings. NO declares receiving travel grants from MSD, Pfizer and Sanofi.

## Appendix A Supplementary data

Appendix

Study Group Members List

Pegasus Protocol
