# Supplementary material for: Paediatric invasive group A streptococcal infections and associations with viral infections in 15 European countries after lifting non-pharmaceutical interventions against SARS-CoV-2: an interrupted time-series analysis
Source: Lancet Reg Health Eur. 2025 Oct 18;59:101497. doi: 10.1016/j.lanepe.2025.101497 (PMC12569802; doi:10.1016/j.lanepe.2025.101497)
Supplement: Appendix [file mmc1.docx]

APPENDIX LIST

[Appendix 1: List of participating centres 2](#_Toc207647748)

[Appendix 2: Evolution of the stringency index across countries (N=15) 4](#_Toc207647749)

[Appendix 3: Evolution of the relative percentage of google search for the *topics* “chickenpox”, “flu”, and “bronchiolitis” between January 1^st^ 2018 and March 31^st^ 2024 in the 15 participating countries. 5](#_Toc207647750)

[Appendix 4: Clinical phenotypes 8](#_Toc207647751)

[Appendix 5: Correlograms and residuals of the main analysis 9](#_Toc207647752)

[Appendix 6: Evolution of the monthly number of iGAS cases by age categories from January 1^st^ 2018 to March 31^st^ 2024 assessed by interrupted time series analyses. a: under two-years-old (N=911), b: between two and five-years-old (N=577), c: above five-years-old (N=324 10](#_Toc207647753)

[Appendix 7: Subgroup analyses of the overall increase in the monthly number of iGAS cases during the post-NPIs period by countries, age-categories and clinical outcome, N=2003. 10](#_Toc207647754)

[Appendix 8 : Evolution of the monthly number of iGAS cases by severity from January 1^st^ 2018 to March 31^st^ 2024 assessed by interrupted time series analyses. a: iGAS cases admitted to general ward, N=1483, b: iGAS cases admitted to ICU, N=580 12](#_Toc207647755)

[Appendix 9: Evolution of the monthly number of iGAS cases by country from January 1^st^ 2018 to March 31^st^ 2024 assessed by interrupted time series analyses. a: Denmark (N=128), b: France (N=72), c: Latvia (N=198), d: Netherlands (N=541), e: Portugal (N= 179), f: Spain (N = 129), g: Switzerland (N=531), h: United Kingdom (N=160) 13](#_Toc207647756)

[Appendix 10: Number of cases of each clinical phenotype of iGAS in each participating country during the baseline and post-NPIs periods. (N = 15 countries) 15](#_Toc207647757)

[Appendix 11: Paediatric emergency department visits and hospital admissions in 2023 19](#_Toc207647758)

[Appendix 12: Comparison between the incidence of syndromic varicella cases and Google Trends data in the Netherlands and in the United Kingdom 21](#_Toc207647759)

[Appendix 13: Comparison between the syndromic and microbiological influenza national data and Google Trends data in the Netherlands, Spain and Denmark 22](#_Toc207647760)

[Appendix 14: Comparison between the syndromic and microbiological RSV national data and Google Trends data in the Netherlands, Spain and Denmark 24](#_Toc207647761)

[Appendix 15: Pearson correlation tests between Google Trends data and national syndromic or microbiological data from a subset of participating centers. 26](#_Toc207647762)

[Appendix 16: Correlation between the evolution of viruses (Influenza, RSV and varicella) and clinical phenotypes of iGAS. 27](#_Toc207647763)

[Appendix 17: Acknowledgement for participating study groups 29](#_Toc207647764)

# Appendix 1: List of participating centres

| **Country** | **Center** | **Participation** | **Data collection** | **Informed consent** |
| --- | --- | --- | --- | --- |
| Austria | Medical University of Graz, Department of general paediatrics, Graz. | Directly participating in PEGASUS | Pro- and retrospective | Obtained for prospective patients |
| Belgium | CHU Tivoli, Service Pédiatrie, La Louvière, | Existing local data collection | Retrospective | Exempt |
| Denmark | Danish national database | Existing regional/local/national data collection | Pro- and retrospective | Exempt |
| France | Hôpital Universitaire Robert Debré, Department of general paediatrics and paediatric infectious diseases, Paris. | Existing local data collection | Pro- and retrospective | Exempt |
| Greece | P. and A. Kyriakou Children's Hospital, National and Kapodistrian University of Athens (NKUA), Second Dept of Paediatrics, Athens. | Directly participating in PEGASUS | Pro- and retrospective | Exempt |
|  | Aghia Sophia Children's Hospital, National and Kapodistrian University of Athens, First department of paediatrics, Athens. | Directly participating in PEGASUS | Pro- and retrospective | Exempt |
| Iceland | Children’s hospital Iceland, Department of Infectious Diseases, Reykjavik. | Directly participating in PEGASUS | Pro- and retrospective | Exempt |
| Italy | Fondazione Policlinico Universitario A. Gemelli IRCCS, Department of Woman and Child Health and Public Health, Roma. | Directly participating in PEGASUS | Pro- and retrospective | Exempt |
|  | Fondazione IRCCS Policlinico San Matteo, Paediatric Clinic, Pavia. | Existing local data collection | Retrospective | Obtained for all patients as part of a hospital policy |
| Latvia | Children University Hospital Riga, Department of paediatric and infectious diseases, Riga. | Directly participating in PEGASUS | Pro- and retrospective | Exempt |
| The Netherlands | COPP-iGAS consortium | Existing regional data collection | Pro- and retrospective | Obtained for prospective patients |
|  | Northwest Clinics, Department of paediatrics. Alkmaar and Den Helder. | Directly participating in PEGASUS | Pro- and retrospective | Exempt |
|  | Erasmus MC, Department of general paediatrics, Rotterdam. | Directly participating in PEGASUS | Pro- and retrospective | Exempt |
|  | Maasstad Ziekenhuis, Department of paediatrics, Rotterdam. | Directly participating in PEGASUS | Pro- and retrospective | Exempt |
| Poland | Wroclaw Medical University, Department of paediatric Infectious Disease, Wroclaw. | Directly participating in PEGASUS | Pro- and retrospective | Exempt |
| Portugal | Hospital Dona Estefania, Department of paediatrics, Lisbon. | Existing national data collection | Pro- and retrospective | Exempt |
| Slovenia | University Medical Center Ljubljana, Division of Paediatrics, Ljubljana. | Directly participating in PEGASUS | Pro- and retrospective | Exempt |
| Spain | Hospital Universitario La Paz, Department of paediatric infectious diseases, Madrid. | Existing regional data collection | Prospective | Exempt |
|  | Gregorio Marañón Hospital, Department of paediatrics, Madrid. | Existing regional data collection | Prospective | Exempt |
| Switzerland | Swiss Pediatric surveillance unit (SPSU) | Existing national data collection | Prospective | Exempt |
| United Kingdom | St Mary's Hospital - Imperial College NHS Healthcare Trust, Department of Paediatric Emergency Medicine, London. | Directly participating in PEGASUS | Pro- and retrospective | Exempt |
|  | Great North Children's Hospital. Paediatric Immunology & Infectious Diseases. Newcastle-upon-Tyne. | Directly participating in PEGASUS | Pro- and retrospective | Exempt |
|  | Alder Hey Children's Hospital, Department of Infectious Diseases, Liverpool. | Directly participating in PEGASUS | Pro- and retrospective | Exempt |

# Appendix 2: Evolution of the stringency index across countries (N=15)

| **Month** | **Country with a stringency < 40%** | **Cumulative number of countries with a stringency index <40%** |
| --- | --- | --- |
| **May 2021** | Italy | 1/15 |
| **June 2021** | - | 1/15 |
| **July 2021** | - | 1/15 |
| **August 2021** | Denmark, Poland | 3/15 |
| **September 2021** | - | 3/15 |
| **October 2021** | - | 3/15 |
| **November 2021** | - | 3/15 |
| **December 2021** | - | 3/15 |
| **January 2022** | Portugal, Spain | 5/15 |
| **February 2022** | United Kingdom | 6/15 |
| **March 2022** | Austria, France, Latvia, Netherlands, Slovenia, Switzerland | 12/15 |
| **April 2022** | - | 12/15 |
| **May 2022** | Greece | 13/15 |
| **June 2022** | Iceland | 14/15 |
| **July 2022** | - | 14/15 |
| **August 2022** | - | 14/15 |
| **September 2022** | - | 14/15 |
| **October 2022** | Belgium | 15/15 |

To define the dates of the different study periods, we used the *stringency index* developed by Our World In Data^15^ during the pandemic. This index is a composite measure based on nine response indicators including school closures, workplace closures and travel bans, rescaled to a value from 0 to 100 (100 = strictest). By March 2022, most of the participating countries (12/15) had a low stringency index (<40% for the total population weighted according to vaccination status rules).

# Appendix 3: Evolution of the relative percentage of google search for the *topics* “chickenpox”, “flu”, and “bronchiolitis” between January 1^st^ 2018 and March 31^st^ 2024 in the 15 participating countries.

1.
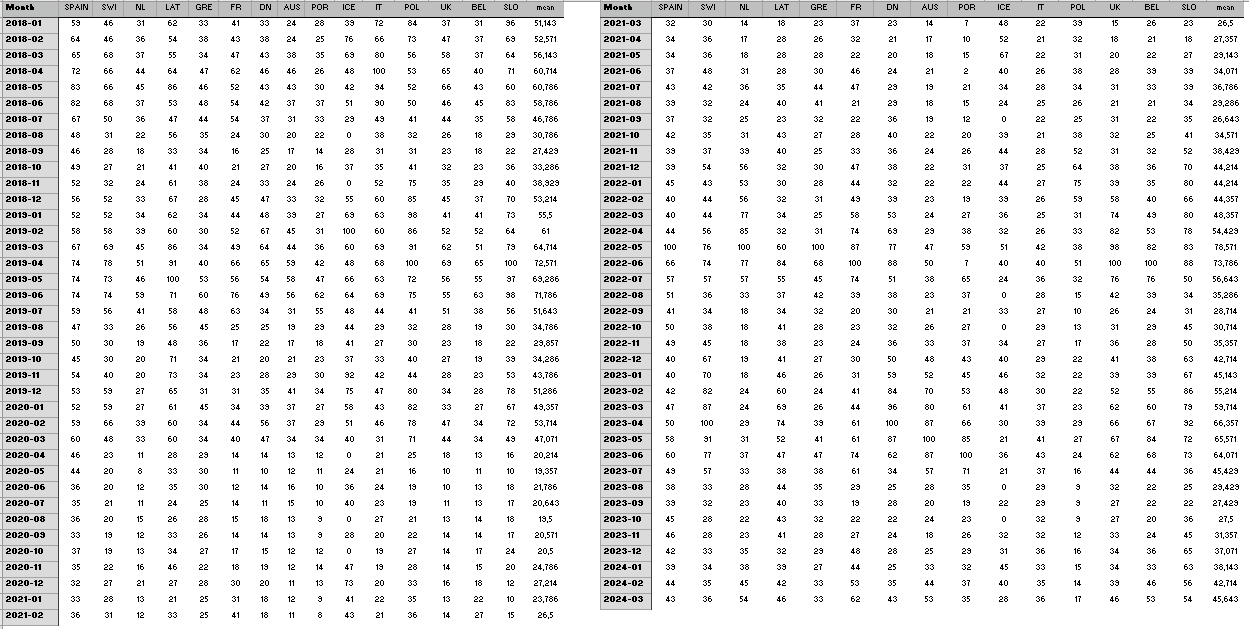
Chickenpox
2.
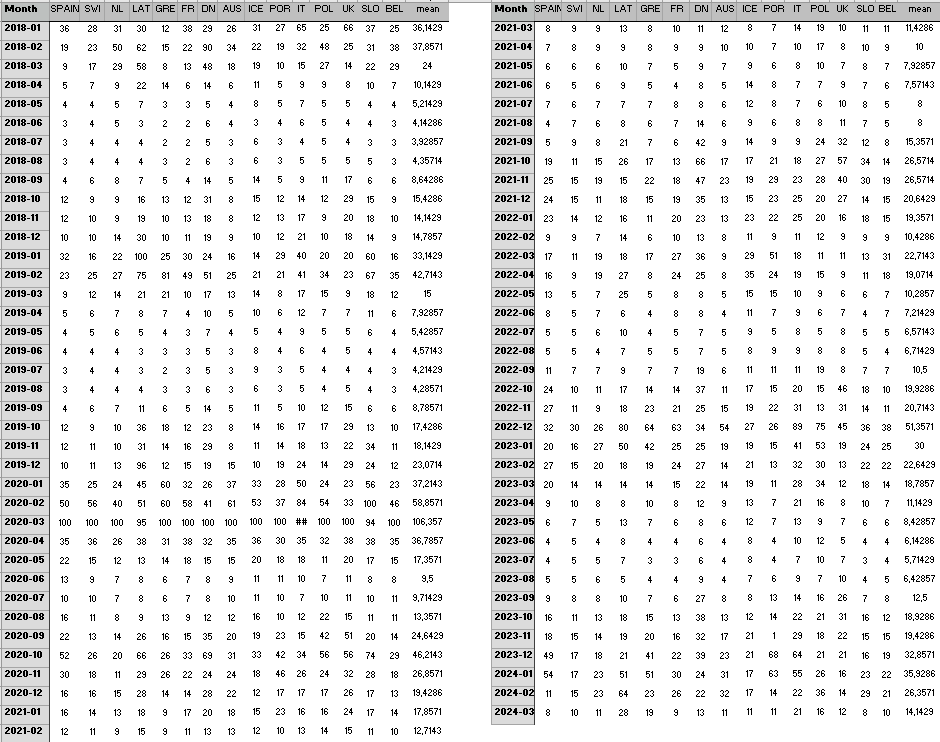
Flu
3.
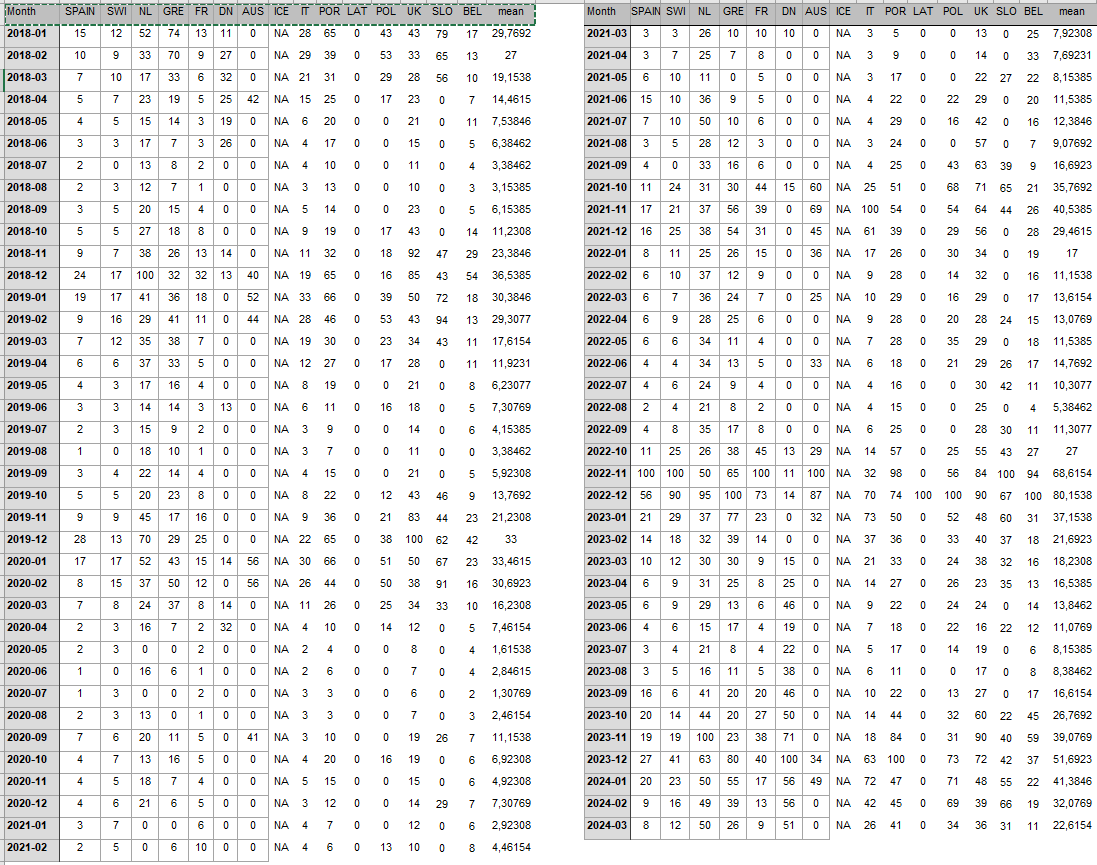
Bronchiolitis

# Appendix 4: Clinical phenotypes

For several clinical phenotypes, composite categories were used.

Sepsis was defined as one or more of the following clinical phenotypes: bacteraemia, sepsis, septic shock, or streptococcal toxic shock syndrome.

Bone & joint infection was defined as one or more of the following: arthritis, osteomyelitis or pyomyositis.

SSTI was defined as one or more of the following: cellulitis, erysipelas, necrotizing fasciitis, and skin abscess. The variable skin abscess was collected as a separate category in the PEGASUS data collection and in Portugal.

Pneumonia was defined as pneumonia with or without empyema

ENT or abscess was defined as any ENT infection, e.g. mastoiditis, ENT abscess (PEGASUS, Portugal, Spain, Switzerland), any abscess (Dutch Consortium, Portugal, Switzerland), other abscess (PEGASUS) and lymphadenitis (PEGASUS, COPP, Denmark, Spain)

| **Clinical Phenotypes** | **Number (percentage)** | **Number (percentage) of STSS for this phenotype** |
| --- | --- | --- |
| Bacteriemia and sepsis cases including STSS | 796 (38.1) | 93 (11.7) |
| STSS cases | 93 (4.4) |  |
| ENT and abscess cases | 535 (25.6); Missing: 128 (6.1) | 6 (1.1) |
| SSTI cases including NF | 429 (20.5) | 13 (3.0) |
| Necrotizing fasciitis | 84 (4.0) | 3 (3.6) |
| Pneumonia cases | 475 (22.7) | 32 (6.7) |
| Osteo-arthritis cases | 337 (16.1) | 10 (3.0) |
| Meningitis cases | 82 (3.9) | 2 (2.4) |
| Other  Patients with only diagnosis “other”  Patients with no clinical phenotype | 336 (16.1)  126 (6.0)  0 (0.0) | 11 (3.3) |

Abbreviations: STSS: Streptococcal Toxic Shock Syndrome; ENT: Ear-Nose-Throat; SSTI : Skin and Soft-Tissue infections. NF: Necrotizing fasciitis.

#

# Appendix 5: Correlograms and residuals of the main analysis


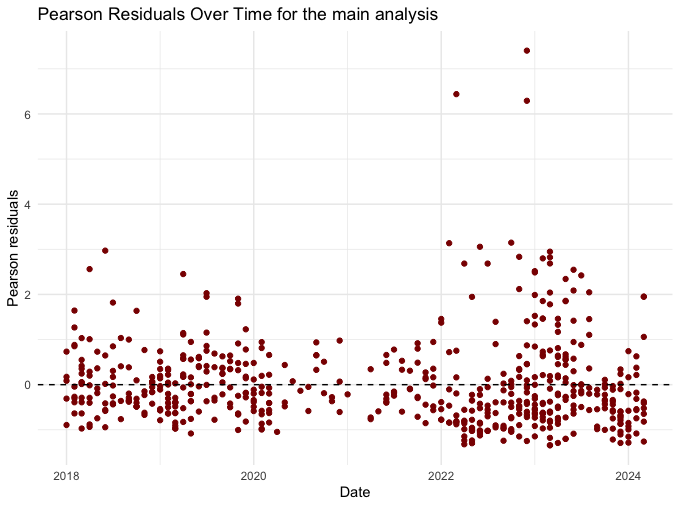

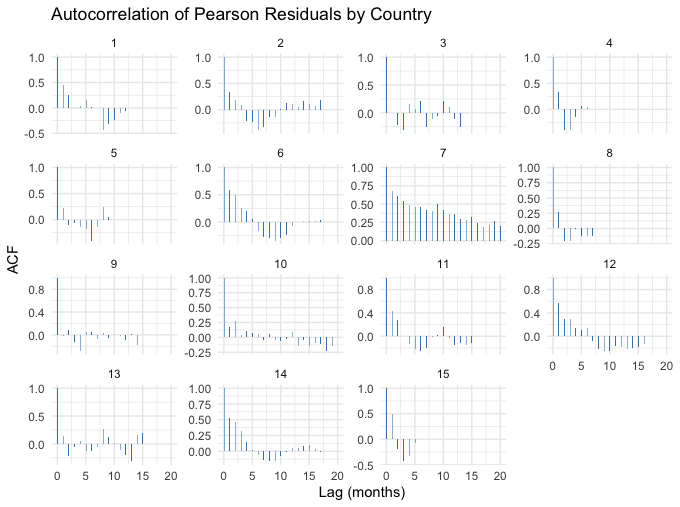


# Appendix 6: Evolution of the monthly number of iGAS cases by age categories from January 1^st^ 2018 to March 31^st^ 2024 assessed by interrupted time series analyses. a: under two-years-old (N=911), b: between two and five-years-old (N=577), c: above five-years-old (N=324


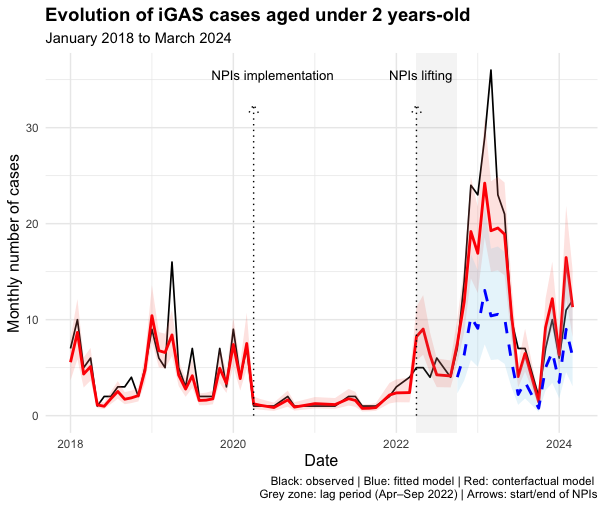

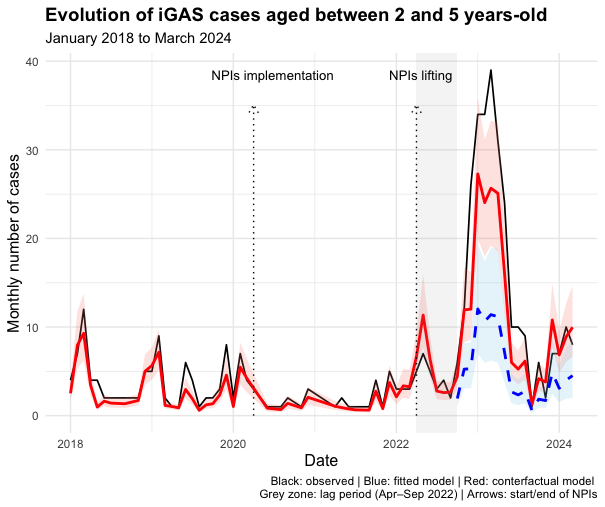


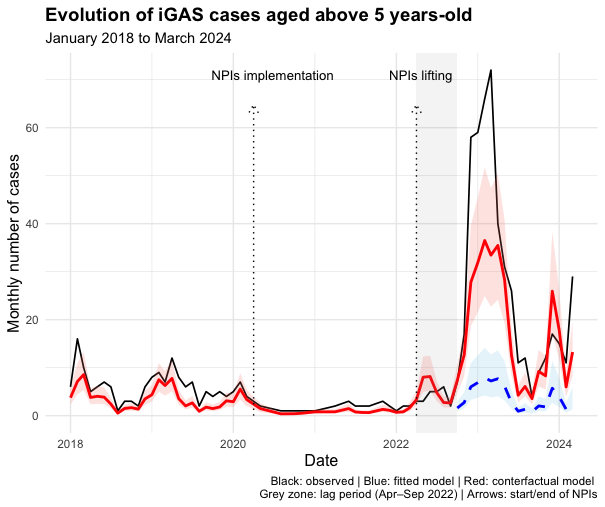


For the study, we defined three study periods: the baseline period: from January 1^st^, 2018, to March 31^st^, 2020, the NPIs period from April 1^st^, 2020, to March 31^st^, 2022, and the post-NPIs period: from October 1^st^, 2022, to March 31^st^, 2024. The effect of the implementation of NPIs was considered immediate, while the effect of their lifting was considered delayed and not expected to be immediate. Thus, we defined a lag period of 6 months from April 1st to September 30th, 2022, highlighted in grey.

The black line corresponds to the observed monthly number of iGAS cases. The red line corresponds to the fitted value of the monthly number of cases associated to the 95% confidence interval. The dashed blue line and its 95% confidence interval corresponds to the expected number of iGAS cases during the post-NPIs period, if no NPIs had ever been implemented.

*Abbreviation : NPIs : Non-pharmaceutical interventions.*

# Appendix 7: Subgroup analyses of the overall increase in the monthly number of iGAS cases during the post-NPIs period by countries, age-categories and clinical outcome, N=2003.

|  | **Overall increase during post-NPIs period*** | **95% CI** |
| --- | --- | --- |
| Cases aged under 2-years-old | + 95.0 | 10.0 - 212.1 |
| Cases aged between 2 and 5 years-old | + 143.8 | 47.1 - 271.1 |
| Cases aged above 5-years-old | + 397.2 | 188.1 - 693.3 |
| General ward admission | + 174.2 % | 98.0 - 270.0 |
| ICU admission | + 271.2 % | 111.9 - 478.4 |

|  | **Overall increase during post-NPIs period*** | **95% CI** |
| --- | --- | --- |
| Denmark** | + 315.6 % | 84.9 – 546.2 |
| France** | + 391.7 % | -40.2 – 627.5 |
| Latvia** | + 222.6 % | 37.8 – 407.5 |
| Netherlands** | + 340.1 % | 66.6 – 613.6 |
| Portugal** | + 310.1% | 49.1 – 571.1 |
| Spain** | + 246.1 % | -34.9 – 527.0 |
| Switzerland** | + 451.7 % | 228.9 – 674.4 |
| UK** | + 159.0 % | -32.9 – 351.0 |

* For the study, we defined three study periods: the baseline period: from January 1st, 2018, to March 31st 2020, the NPIs period from April 1st 2020 to March 31st 2022 and the post-NPIs period: from October 1st 2022 to March 31st 2024. The effect of the implementation of NPIs was considered immediate, while the effect of their lifting was considered delayed and not expected to be immediate. Thus, we defined a lag period of 6 months from April 1st to September 30th 2022

The overall increase is calculated as the overall difference between the modulization of the outcome during the post-NPIs period and the counterfactual scenario – if no NPIs had been implemented – accounting for the trend of the pre-NPIs period.

**Analyses were performed for countries with more than 50 iGAS cases over the study period. To investigate country-specific effects and address overdispersion more directly within each context, separate quasi-Poisson models were fitted for each country.

*Abbreviations: NPIs: non-pharmaceutical interventions; iGAS: invasive Group A streptococcus; ENT: Ear–Nose-Throat*

# Appendix 8 : Evolution of the monthly number of iGAS cases by severity from January 1^st^ 2018 to March 31^st^ 2024 assessed by interrupted time series analyses. a: iGAS cases admitted to general ward, N=1483, b: iGAS cases admitted to ICU, N=580


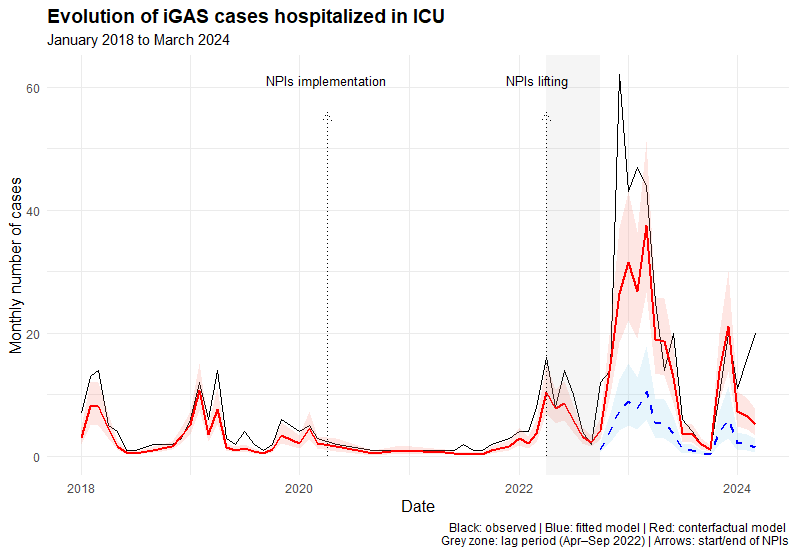

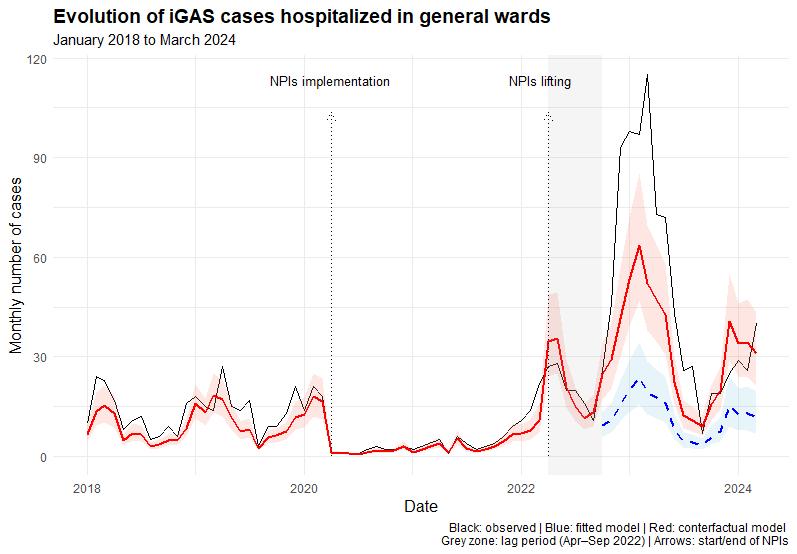


For the study, we defined three study periods: the baseline period: from January 1^st^, 2018, to March 31^st^, 2020, the NPIs period from April 1^st^, 2020, to March 31^st^, 2022, and the post-NPIs period: from October 1^st^, 2022, to March 31^st^, 2024. The effect of the implementation of NPIs was considered immediate, while the effect of their lifting was considered delayed and not expected to be immediate. Thus, we defined a lag period of 6 months from April 1st to September 30th, 2022, highlighted in grey.

The black line corresponds to the observed monthly number of iGAS cases. The red line corresponds to the fitted value of the monthly number of cases associated to the 95% confidence interval. The dashed blue line and its 95% confidence interval corresponds to the expected number of iGAS cases during the post-NPIs period, if no NPIs had ever been implemented.

*Abbreviation : NPIs : Non-pharmaceutical interventions, ICU: Intensive care unit*

# Appendix 9: Evolution of the monthly number of iGAS cases by country from January 1^st^ 2018 to March 31^st^ 2024 assessed by interrupted time series analyses. a: Denmark (N=128), b: France (N=72), c: Latvia (N=198), d: Netherlands (N=541), e: Portugal (N= 179), f: Spain (N = 129), g: Switzerland (N=531), h: United Kingdom (N=160)

**a.** **b.**


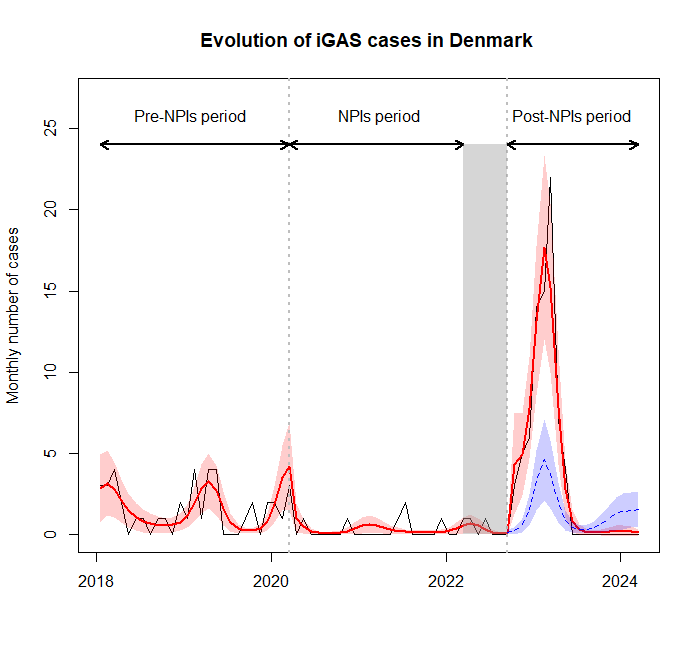

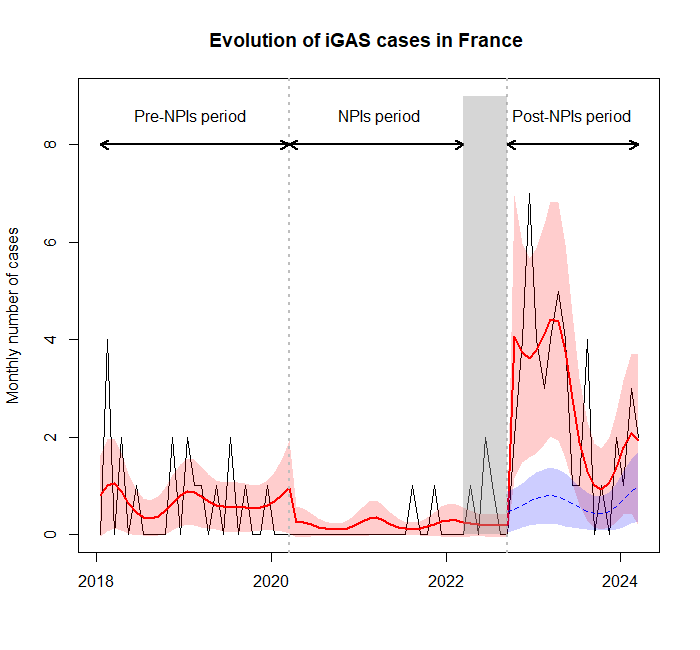


**c.** **d.**


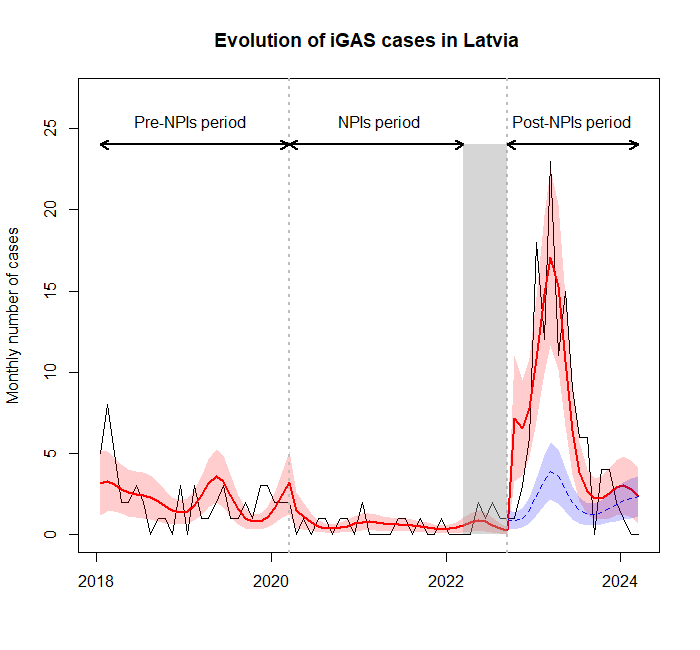

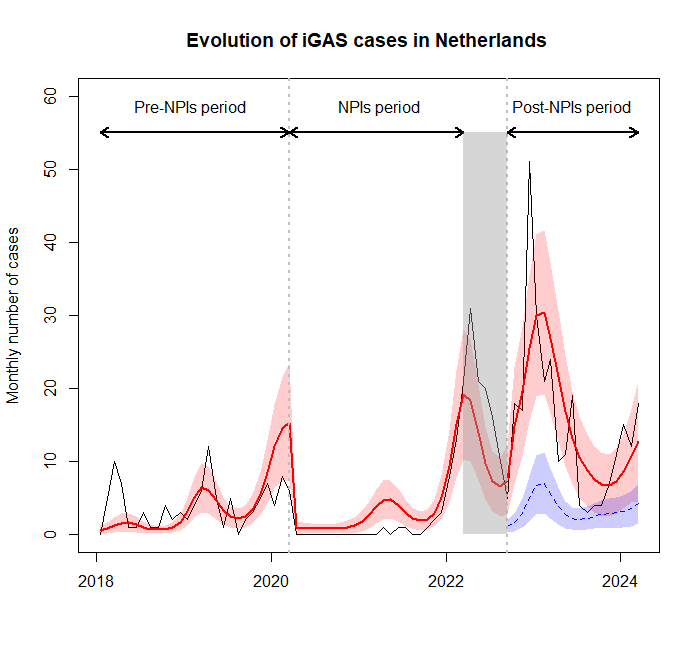


**e.** **f.**


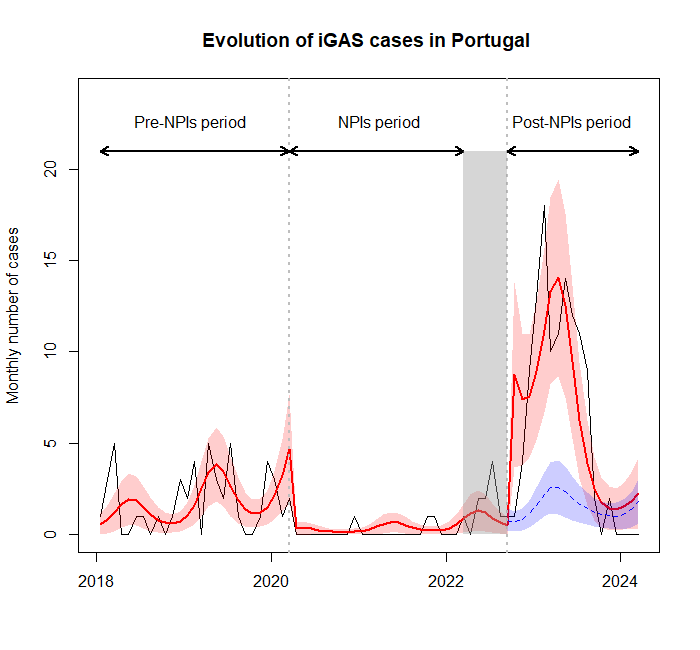

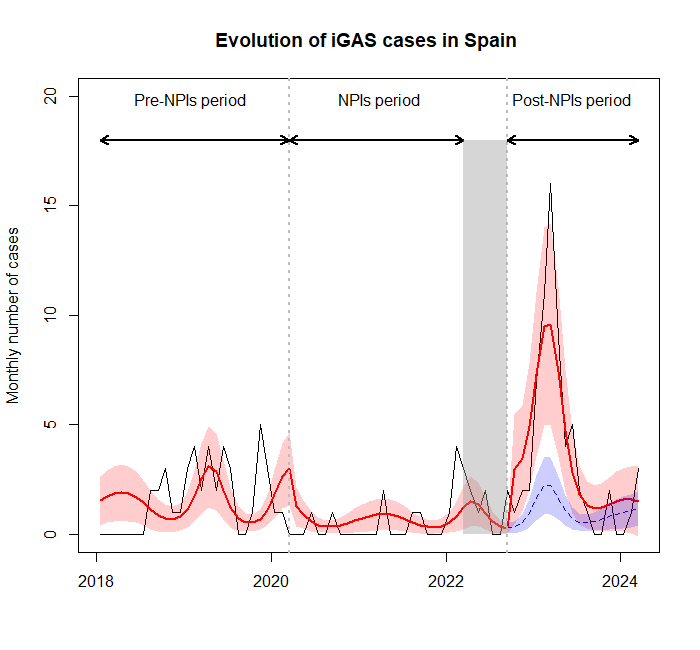


**g.** **h.**


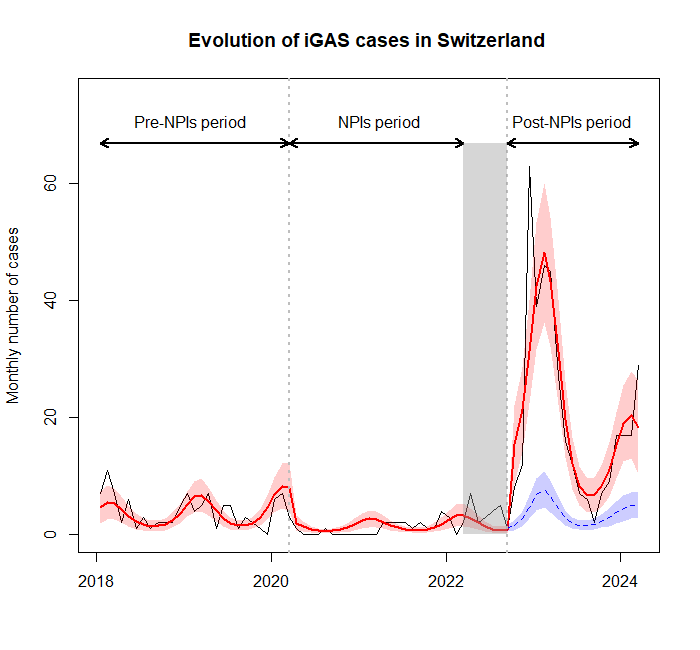

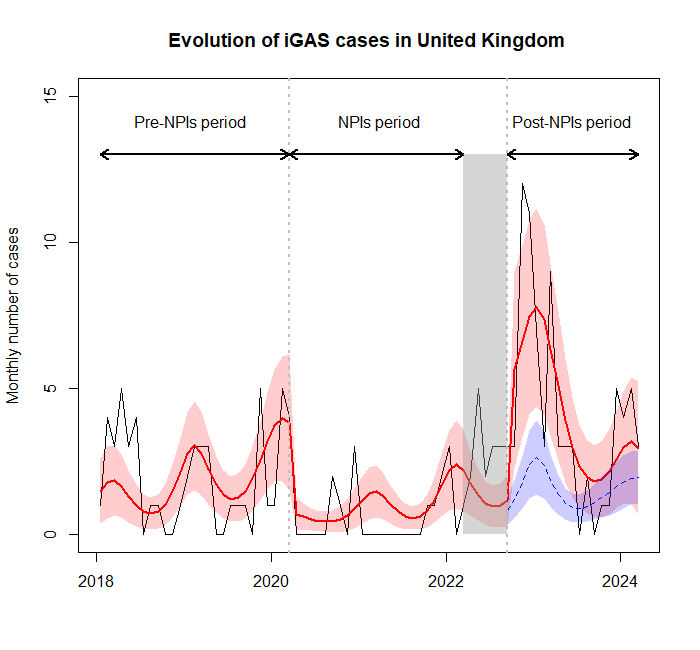


For the study, we defined three study periods: the baseline period: from January 1^st^, 2018, to March 31^st^, 2020, the NPIs period from April 1^st^, 2020, to March 31^st^, 2022, and the post-NPIs period: from October 1^st^, 2022, to March 31^st^, 2024. The effect of the implementation of NPIs was considered immediate, while the effect of their lifting was considered delayed and not expected to be immediate. Thus, we defined a lag period of 6 months from April 1st to September 30th, 2022, highlighted in grey.

The black line corresponds to the observed monthly number of iGAS cases. The red line corresponds to the fitted value of the monthly number of cases associated to the 95% confidence interval. The dashed blue line and its 95% confidence interval corresponds to the expected number of iGAS cases during the post-NPIs period, if no NPIs had ever been implemented.
The main multilevel model was used for secondary analysis based on multi-country data. However, to investigate country-specific effects and address overdispersion more directly within each context, separate quasi-Poisson models were fitted for each country.

*Abbreviation: NPIs: Non-pharmaceutical intervention*

# Appendix 10: Number of cases of each clinical phenotype of iGAS in each participating country during the baseline and post-NPIs periods. (N = 15 countries)

*The baseline period is defined as January 1st, 2018, to March 31st, 2020.*

*The NPIs period is defined as April 1st, 2020, to March 31st, 2022.*

*The post-NPIs period is defined as: April 1st, 2022, to March 31st, 2024.*

| **AUSTRIA** | **Baseline period, N (%)** | **NPI period, N (%)** | **Post-NPIs period N (%)** |
| --- | --- | --- | --- |
| All clinical phenotypes | 7 | 3 | 12 |
| Bacteriemia and sepsis cases including STSS | 1 (14.3) | 0 (0.0) | 3 (25.0) |
| ENT and abscess cases | 2 (28.6) | 1 (33.3) | 5 (41.7) |
| SSTI cases including necrotizing fasciitis | 1 (14.3) | 0 (0.0) | 0 (0.0) |
| Pneumonia cases | 0 (0.0) | 0 (0.0) | 1 (8.3) |
| Osteo-arthritis cases | 3 (42.9) | 3 (100.0) | 3 (25.0) |
| Meningitis cases | 0 (0.0) | 0 (0.0) | 0 (0.0) |

| **BELGIUM** | **Baseline period, N (%)** | **NPI period, N (%)** | **Post-NPIs period N (%)** |
| --- | --- | --- | --- |
| All clinical phenotypes | 1 | 2 | 3 |
| Bacteriemia and sepsis cases including STSS | 0 (0.0) | 1 (50.0) | 2 (66.6) |
| ENT and abscess cases | 0 (0.0) | 0 (0.0) | 0 (0.0) |
| SSTI cases including necrotizing fasciitis | 0 (0.0) | 0 (0.0) | 1 (33.3) |
| Pneumonia cases | 1 (100) | 0 (0.0) | 0 (0.0) |
| Osteo-arthritis cases | 0 (0.0) | 0 (0.0) | 1 (33.3) |
| Meningitis cases | 0 (0.0) | 0 (0.0) | 0 (0.0) |

| **DENMARK** | **Baseline period, N (%)** | **NPI period, N (%)** | **Post-NPIs period N (%)** |
| --- | --- | --- | --- |
| All clinical phenotypes | 43 | 7 | 78 |
| Bacteriemia and sepsis cases including STSS | 26 (60.5) | 6 (85.7) | 48 (61.5) |
| ENT and abscess cases | Not available | Not available | Not available |
| SSTI cases including necrotizing fasciitis | 12 (27.9) | 0 (0) | 38 (48.7) |
| Pneumonia cases | 8 (18.6) | 1 (14.3) | 24 (30.8) |
| Osteo-arthritis cases | 8 (18.6) | 4 (57.1) | 15 (19.2) |
| Meningitis cases | 5 (11.6) | 1 (14.3) | 6 (7.7) |

| **FRANCE** | **Baseline period, N (%)** | **NPI period, N (%)** | **Post-NPIs period N (%)** |
| --- | --- | --- | --- |
| All clinical phenotypes | 18 | 2 | 52 |
| Bacteriemia and sepsis cases including STSS | 9 (50.0) | 1 (50.0) | 10 (19.2) |
| ENT and abscess cases | 1 (5.6) | 0 (0) | 12 (23.1) |
| SSTI cases including necrotizing fasciitis | 1 (5.6) | 0 (0) | 2 (3.8) |
| Pneumonia cases | 4 (22.2) | 0 (0) | 13 (25.0) |
| Osteo-arthritis cases | 10 (55.6) | 1 (50.0) | 19 (36.5) |
| Meningitis cases | 0 | 0 (0) | 1 (1.9) |

| **GREECE** | **Baseline period, N (%)** | **NPI period, N (%)** | **Post-NPIs period N (%)** |
| --- | --- | --- | --- |
| All clinical phenotypes | 7 | 1 | 34 |
| Bacteriemia and sepsis cases including STSS | 5 (71.4) | 1 (100.0) | 23 (67.6) |
| ENT and abscess cases | 1 (14.3) | 0 (0.0) | 8 (23.5) |
| SSTI cases including necrotizing fasciitis | 0 (0.0) | 0 (0.0) | 8 (23.5) |
| Pneumonia cases | 1 (14.3) | 0 (0.0) | 3 (8.8) |
| Osteo-arthritis cases | 0 (0.0) | 1 (100.0) | 3 (8.8) |
| Meningitis cases | 0 (0.0) | 0 (0.0) | 0 (0.0) |

| **ICELAND** | **Baseline period, N (%)** | **NPI period, N (%)** | **Post-NPIs period N (%)** |
| --- | --- | --- | --- |
| All clinical phenotypes | 0 | 0 | 17 |
| Bacteriemia and sepsis cases including STSS | 0 (0.0) | 0 (0.0) | 5 (29.4) |
| ENT and abscess cases | 0 (0.0) | 0 (0.0) | 2 (11.8) |
| SSTI cases including necrotizing fasciitis | 0 (0.0) | 0 (0.0) | 3 (17.6) |
| Pneumonia cases | 0 (0.0) | 0 (0.0) | 10 (58.8) |
| Osteo-arthritis cases | 0 (0.0) | 0 (0.0) | 2 (11.8) |
| Meningitis cases | 0 (0.0) | 0 (0.0) | 1 (5.9) |

| **ITALY** | **Baseline period, N (%)** | **NPI period, N (%)** | **Post-NPIs period N (%)** |
| --- | --- | --- | --- |
| All clinical phenotypes | 3 | 0 | 9 |
| Bacteriemia and sepsis cases including STSS | 1 (33.3) | 0 (0.0) | 3 (33.3) |
| ENT and abscess cases | 1 (33.3) | 0 (0.0) | 2 (22.2) |
| SSTI cases including necrotizing fasciitis | 0 (0.0) | 0 (0.0) | 1 (11.1) |
| Pneumonia cases | 1 (33.3) | 0 (0.0) | 4 (44.4) |
| Osteo-arthritis cases | 0 (0.0) | 0 (0.0) | 0 |
| Meningitis cases | 1 (33.3) | 0 (0.0) | 2 (22.2) |

| **LATVIA** | **Baseline period, N (%)** | **NPI period, N (%)** | **Post-NPIs period N (%)** |
| --- | --- | --- | --- |
| All clinical phenotypes | 59 | 11 | 128 |
| Bacteriemia and sepsis cases including STSS | 2 (3.4) | 1 (9.1) | 4 (3.1) |
| ENT and abscess cases | 31 (52.5) | 7 (63.6) | 72 (56.3) |
| SSTI cases including necrotizing fasciitis | 9 (15.3) | 5 (45.5) | 24 (18.8) |
| Pneumonia cases | 0 (0.0) | 0 (0.0) | 4 (3.1) |
| Osteo-arthritis cases | 2 (3.4) | 0 (0.0) | 6 (4.7) |
| Meningitis cases | 0 (0.0) | 0 (0.0) | 0 (0.0) |

| **NETHERLANDS** | **Baseline period, N (%)** | **NPI period, N (%)** | **Post-NPIs period N (%)** |
| --- | --- | --- | --- |
| All clinical phenotypes | 108 | 51 | 382 |
| Bacteriemia and sepsis cases including STSS | 17 (15.7) | 5 (9.8) | 126 (33.0) |
| ENT and abscess cases | 42 (38.9) | 5 (9.8) | 87 (22.8) |
| SSTI cases including necrotizing fasciitis | 20 (18,5) | 5 (9.8) | 75 (19.6) |
| Pneumonia cases | 13 (12.0) | 7 (13.7) | 107 (28.0) |
| Osteo-arthritis cases | 4 (3.7) | 6 (11.8) | 51 (13.4) |
| Meningitis cases | 1 (0.9) | 0 (0.0) | 26 (6.8) |

| **POLAND** | **Baseline period, N (%)** |  | **Post-NPIs period N (%)** |
| --- | --- | --- | --- |
| All clinical phenotypes | 3 | 0 | 5 |
| Bacteriemia and sepsis cases including STSS | 3 (100) | 0 (0.0) | 5 (100.0) |
| ENT and abscess cases | 0 (0.0) | 0 (0.0) | 1 (20.0) |
| SSTI cases including necrotizing fasciitis | 2 (66.7) | 0 (0.0) | 3 (60.0) |
| Pneumonia cases | 0 (0.0) | 0 (0.0) | 1 (20.0) |
| Osteo-arthritis cases | 1 (33.3) | 0 (0.0) | 0 (0.0) |
| Meningitis cases | 0 (0.0) | 0 (0.0) | 1 (20.0) |

| **PORTUGAL** | **Baseline period, N (%)** | **NPI period, N (%)** | **Post-NPIs period N (%)** |
| --- | --- | --- | --- |
| All clinical phenotypes | 49 | 4 | 126 |
| Bacteriemia and sepsis cases including STSS | 32 (65.3) | 3 (75.0) | 87 (69.0) |
| ENT and abscess cases | 0 (0.0) | 0 (0.0) | 0 (0.0) |
| SSTI cases including necrotizing fasciitis | 15 (30.6) | 0 (0.0) | 23 (18.3) |
| Pneumonia cases | 14 (28.6) | 1 (25.0) | 31 (24.6) |
| Osteo-arthritis cases | 10 (20.4) | 1 (25.0) | 31 (24.6) |
| Meningitis cases | 2 (4.1) | 0 (0.0) | 7 (5.6) |

| **SPAIN** | **Baseline period, N (%)** | **NPI period, N (%)** | **Post-NPIs period N (%)** |
| --- | --- | --- | --- |
| All clinical phenotypes | 42 | 14 | 73 |
| Bacteriemia and sepsis cases including STSS | 13 (31.0) | 2 (14.3) | 20 (27.4) |
| ENT and abscess cases | 9 (21.4) | 4 (28.6) | 32 (43.8) |
| SSTI cases including necrotizing fasciitis | 9 (21.4) | 3 (21.4) | 15 (20.5) |
| Pneumonia cases | 7 (16.7) | 4 (28.6) | 10 (13.7) |
| Osteo-arthritis cases | 5 (11.9) | 1 (7.1) | 5 (6.8) |
| Meningitis cases | 0 (0.0) | 0 (0.0) | 1 (5.0) |

| **SLOVENIA** | **Baseline period, N (%)** | **NPI period, N (%)** | **Post-NPIs period N (%)** |
| --- | --- | --- | --- |
| All clinical phenotypes | 12 | 3 | 31 |
| Bacteriemia and sepsis cases including STSS | 1 (8.3) | 2 (66.7) | 3 (9.7) |
| ENT and abscess cases | 11 (8.3) | 1 (33.3) | 10 (32.3) |
| SSTI cases including necrotizing fasciitis | 0 (0.0) | 0 (0.0) | 2 (6.5) |
| Pneumonia cases | 4 (33.3) | 1 (33.3) | 10 (32.3) |
| Osteo-arthritis cases | 3 (25.0) | 0 (0.0) | 10 (32.3) |
| Meningitis cases | 1 (8.3) | 0 (0.0) | 0 (0.0) |

| **SWITZERLAND** | **Baseline period, N (%)** |  | **Post-NPIs period N (%)** |
| --- | --- | --- | --- |
| All clinical phenotypes | 103 | 24 | 401 |
| Bacteriemia and sepsis cases including STSS | 53 (51.5) | 19 (79.2) | 169 (42.1) |
| ENT and abscess cases | 24 (23.3) | 9 (37.5) | 147 (36.7) |
| SSTI cases including necrotizing fasciitis | 11 (10.7) | 4 (16.7) | 106 (26.4) |
| Pneumonia cases | 25 (24.3) | 7 (29.2) | 96 (23.9) |
| Osteo-arthritis cases | 24 (23.3) | 9 (37.5) | 73 (18.2) |
| Meningitis cases | 6 (5.8) | 1 (4.2) | 15 (3.7) |

| **UNITED KINGDOM** | **Baseline period, N (%)** | **NPI period, N (%)** | **Post-NPIs period N (%)** |
| --- | --- | --- | --- |
| All clinical phenotypes | 53 | 14 | 93 |
| Bacteriemia and sepsis cases including STSS | 26 (49.1) | 13 (92.9) | 44 (47.3) |
| ENT and abscess cases | 3 (5.7) | 4 (28.6) | 11 (11.8) |
| SSTI cases including necrotizing fasciitis | 7 (13.2) | 6 (42.9) | 21 (22.6) |
| Pneumonia cases | 15 (28.3) | 4 (28.6) | 43 (46.2) |
| Osteo-arthritis cases | 7 (13.2) | 5 (35.7) | 10 (10.8) |
| Meningitis cases | 2 (3.8) | 0 (0.0) | 2 (2.6) |

*Abbreviations: STSS: Streptococcal Toxic Shock Syndrome; ENT: Ear-Nose-Throat; NPIs: non-pharmaceutical intervention; SSTI : Skin and Soft-Tissue infections.*

# Appendix 11: Paediatric emergency department visits and hospital admissions in 2023

| **Country** | **Center** | **Inclusions in current analysis** | **Emergengy department visits in 2023** | **Hospital admissions in 2023** |
| --- | --- | --- | --- | --- |
| Austria | Medical University of Graz, Department of general paediatrics, Graz. | 22 | 64800 | Approx. 4000 |
| Belgium | CHU Tivoli, Service Pédiatrie, La Louvière, | 6 | 8842 | 1331 |
| Denmark | Danish national database | 128 | Unknown | Unkown |
| France | Hôpital Universitaire Robert Debré, Department of general paediatrics and paediatric infectious diseases, Paris. | 72 | 76648 | 8163 |
| Greece | P. and A. Kyriakou Children's Hospital, National and Kapodistrian University of Athens (NKUA), Second Dept of Paediatrics, Athens. | 11 | 31000 | 6500 |
|  | Aghia Sophia Children's Hospital, National and Kapodistrian University of Athens, First department of paediatrics, Athens. | 31 | 68784 | 13699 |
| Iceland | Children’s hospital Iceland, Department of Infectious Diseases, Reykjavik. | 17 | 18033 | 1654 |
| Italy | Fondazione Policlinico Universitario A. Gemelli IRCCS, Department of Woman and Child Health and Public Health, Roma. | 7 | 15000 | 1000 |
|  | Fondazione IRCCS Policlinico San Matteo, Paediatric Clinic, Pavia. | 5 | 13389 | 1029 |
| Latvia | Children University Hospital Riga, Department of paediatric and infectious diseases, Riga. | 198 | 31820 | 14823 |
| The Netherlands | COPP-iGAS consortium | 453 | Unkown | Unknown |
|  | Northwest Clinics, Department of paediatrics. Alkmaar and Den Helder. | 29 | 7628 | 2028 |
|  | Erasmus MC, Department of general paediatrics, Rotterdam. | 41 | 5049 | 1881 |
|  | Maasstad Ziekenhuis, Department of paediatrics, Rotterdam. | 18 | 6589 | 1844 |
| Poland | Wroclaw Medical University, Department of paediatric Infectious Disease, Wroclaw. | 8 | Approx. 14000 | Approx. 2000 |
| Portugal | Hospital Dona Estefania, Department of paediatrics, Lisbon. | 179 | 68379 | 2868 |
| Slovenia | University Medical Center Ljubljana, Division of Paediatrics, Ljubljana. | 46 | 5922 | 3410 |
| Spain | Hospital Universitario La Paz, Department of paediatric infectious diseases, Madrid. | 50 | 48900 | 3667 |
|  | Gregorio Marañón Hospital, Department of paediatrics, Madrid. | 79 | 57193 | 6384 |
| Switzerland | Swiss Pediatric surveillance unit (SPSU) | 531 | Unknown | Unknown |
| United Kingdom | St Mary's Hospital - Imperial College NHS Healthcare Trust, Department of Paediatric Emergency Medicine, London. | 29 | 21272 | 2178 |
|  | Great North Children's Hospital. Paediatric Immunology & Infectious Diseases. Newcastle-upon-Tyne. | 54 | 40893 | 9448 |
|  | Alder Hey Children's Hospital, Department of Infectious Diseases, Liverpool. | 77 | 66377 | 11208 |

# Appendix 12: Comparison between the incidence of syndromic varicella cases and Google Trends data in the Netherlands and in the United Kingdom


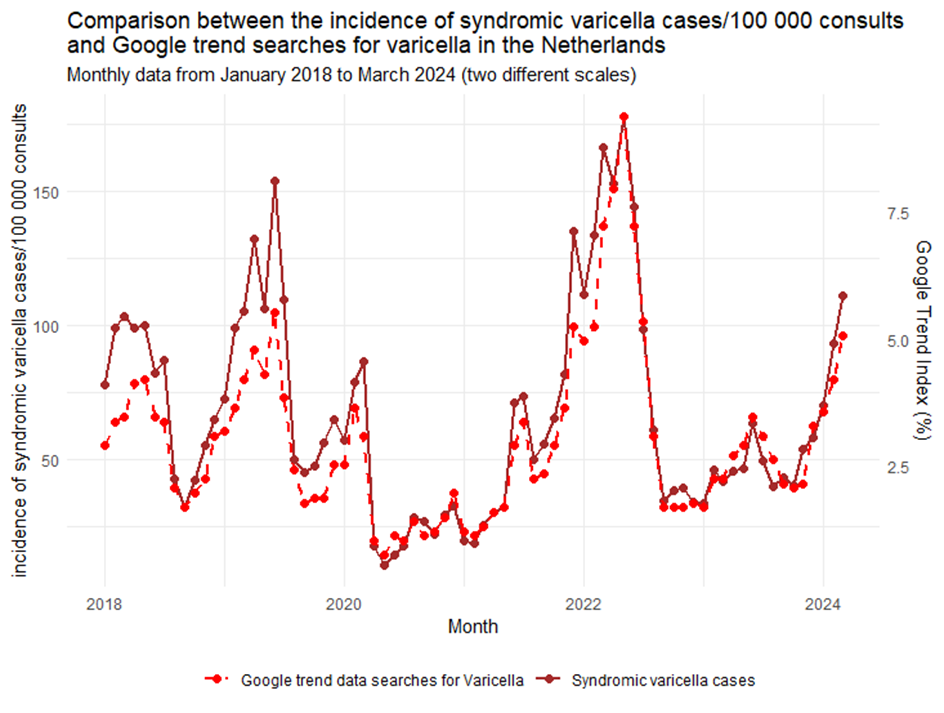


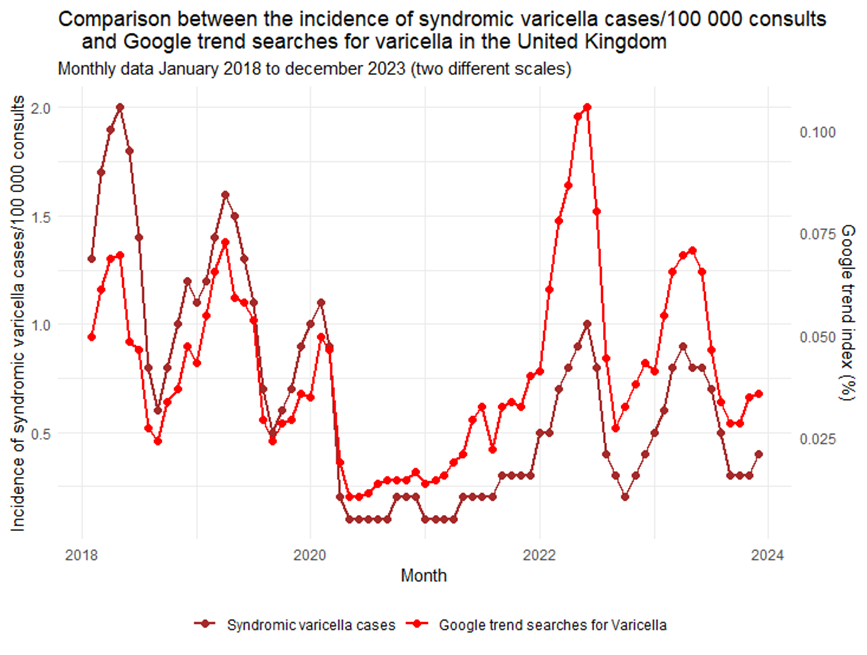


# Appendix 13: Comparison between the syndromic and microbiological influenza national data and Google Trends data in the Netherlands, Spain and Denmark


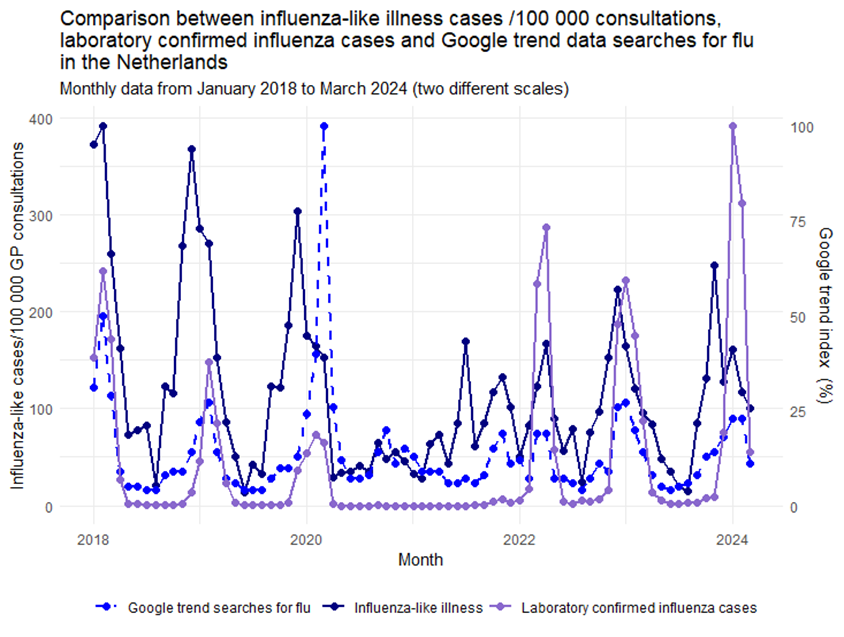


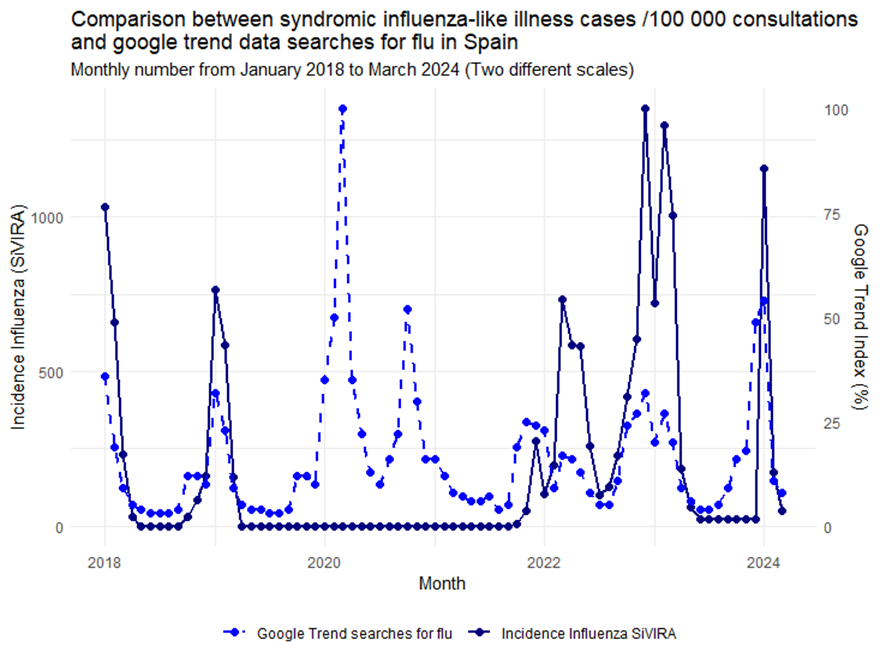


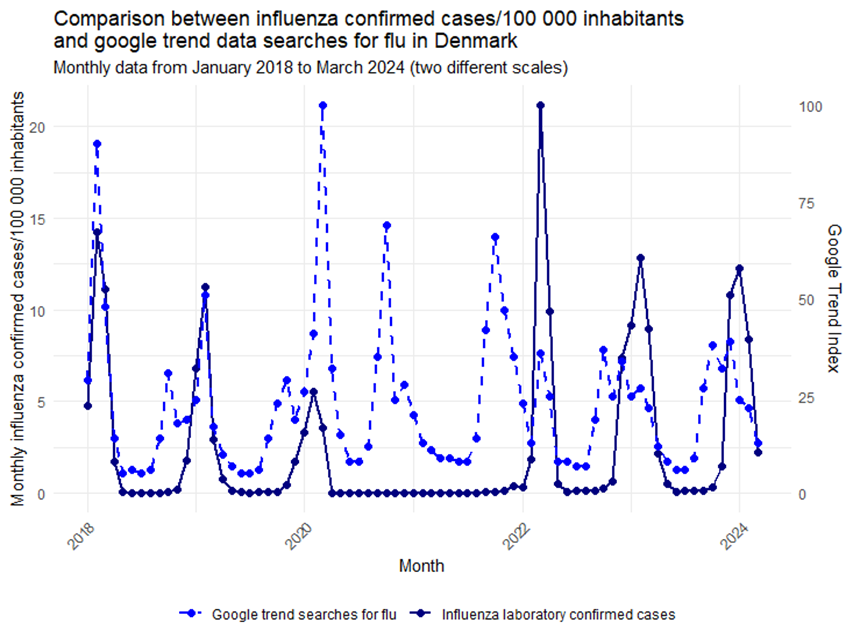


# Appendix 14: Comparison between the syndromic and microbiological RSV national data and Google Trends data in the Netherlands, Spain and Denmark


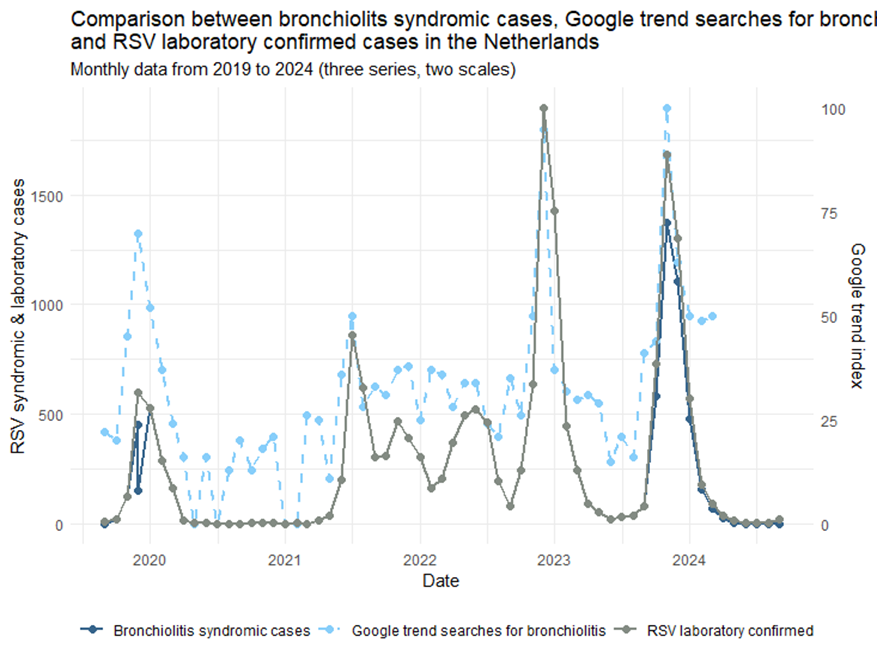


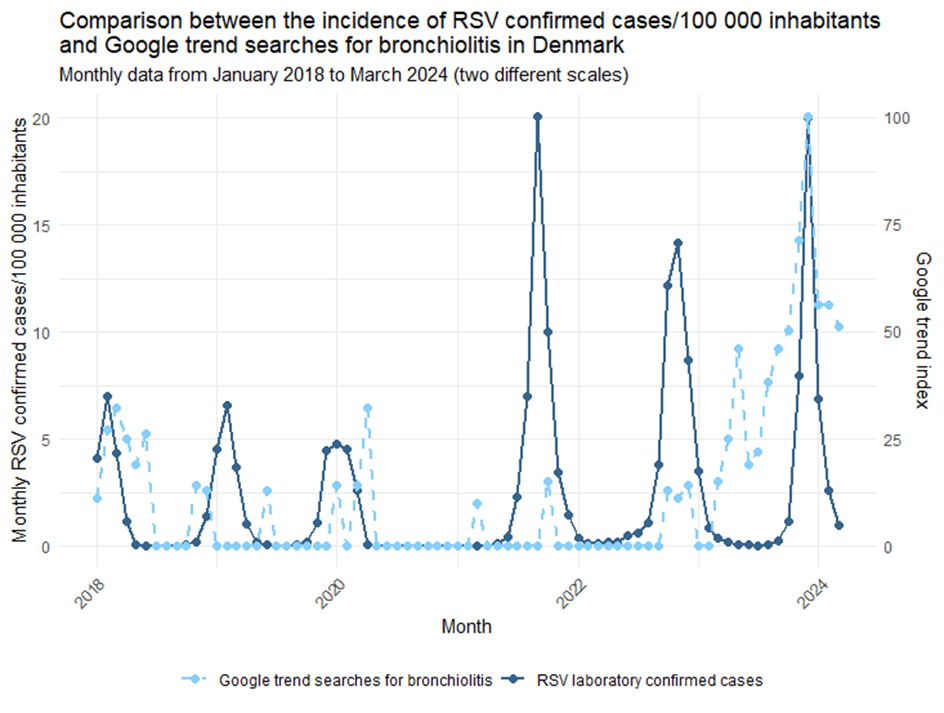


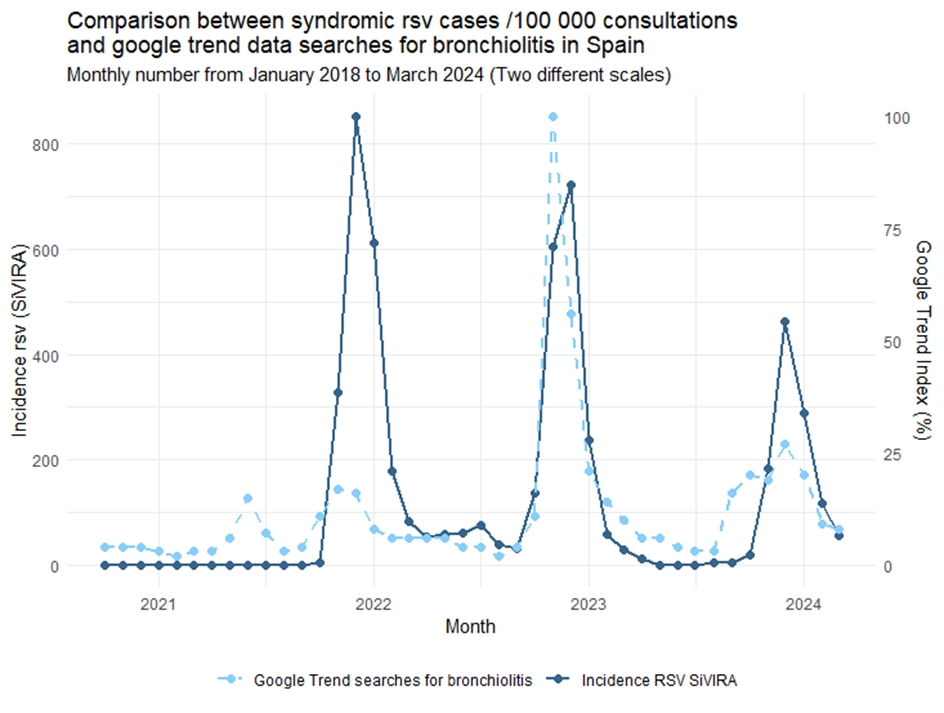


# Appendix 15: Pearson correlation tests between Google Trends data and national syndromic or microbiological data from a subset of participating centers.

| **Google Trends data** | **National data** | **Pearson’s correlation**  **95%CI** | **p-value** |
| --- | --- | --- | --- |
| Varicella in the Netherlands | Syndromic surveillance of varicella in the Netherlands | 0.95  0.92-0.97 | <0.001 |
| Varicella in the UK | Syndromic surveillance of varicella in the UK | 0.66  0.51-0.77 | <0.001 |
| Flu in the Netherlands | Syndromic flu in the Netherlands (excluding the NPIs period*) | 0.69  0.49-0.82 | <0.001 |
| Flu in the Netherlands | Laboratory confirmed cases of Influenza in the Netherlands (excluding the NPIs period*) | 0.38  0.09-0.61 | 0.01 |
| Flu in Spain | Syndromic and laboratory confirmed cases of influenza in Spain (excluding the NPIs period*) | 0.58  0.33-0.75 | <0.001 |
| Flu in Denmark | Laboratory confirmed Influenza in Denmark (excluding the NPIs period*) | 0.69  0.49-0.82 | <0.001 |
| Bronchiolitis in the Netherlands | Syndromic bronchiolitis cases in the Netherlands | 0.73  0.58-0.83 | <0.001 |
| Bronchiolitis in Denmark | Laboratory confirmed RSV in Denmark | 0.34  0.12-0.52 | 0.002 |
| Bronchiolitis in Spain | Syndromic bronchiolitis and confirmed cases of RSV in Spain | 0.64  0.42-0.79 | <0.001 |

# Appendix 16: Correlation between the evolution of viruses (Influenza, RSV and varicella) and clinical phenotypes of iGAS.

| **Correlation between:** | **ENT and abscesses cases** | | | **Osteo-articular cases** | | | **Bacteriemia cases** | | |
| --- | --- | --- | --- | --- | --- | --- | --- | --- | --- |
|  | Rho | 95%CI | p-value | Rho | 95%CI | p-value | Rho | 95%CI | p-value |
| **Influenza** | 0.21 | -0.26-0.61 | 0.37 | 0.48 | 0.04-0.77 | **0.04** | 0.48 | 0.04-0.77 | **0.03** |
| **RSV** | 0.007 | -0.44-0.46 | 0.97 | 0.23 | -0.24-0.63 | 0.32 | 0.29 | -0.18-0.66 | 0.22 |
| **VZV** | 0.76 | 0.47-0.90 | **<0.001** | 0.57 | 0.16-0.82 | **0.009** | 0.62 | 0.23-0.84 | **0.004** |


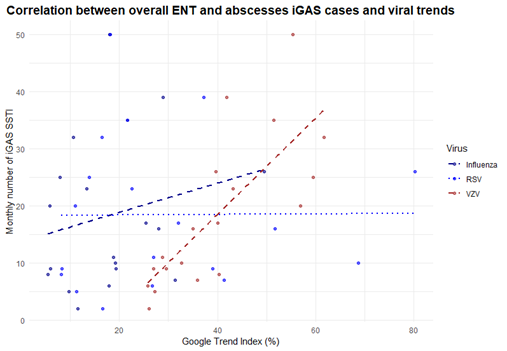

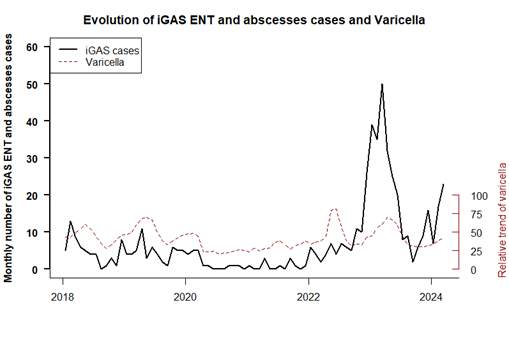


**a**: Graphical representation of the Pearson correlation between the monthly number of iGAS ENT and abscesses cases and the relative trend of VZV during the post NPIs period.

**b**: Evolution of iGAS ENT and abscesses cases (N=534), Influenza and RSV from January 1^st^, 2018, to March 31^st^, 2024


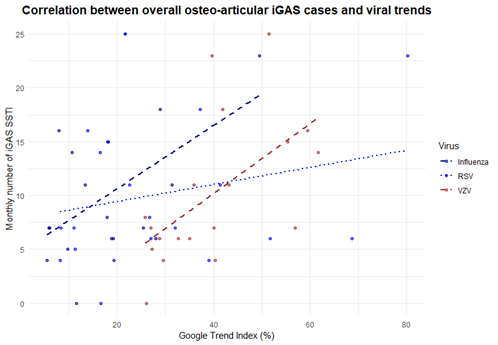

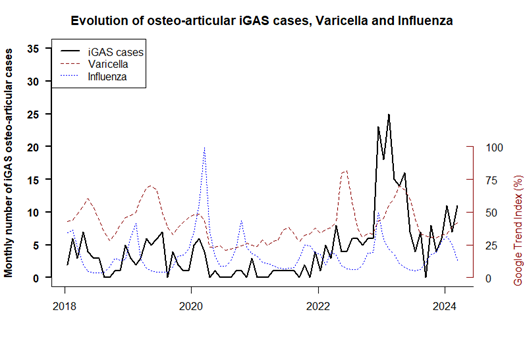


**c**: Graphical representation of the Pearson correlation between the monthly number of osteo-articular iGAS cases and the relative trend of viruses (Influenza and VZV) during the post NPIs period.

d: Evolution of osteo-articular iGAS cases (N=337) and VZV from January 1st, 2018, to March 31st, 2024


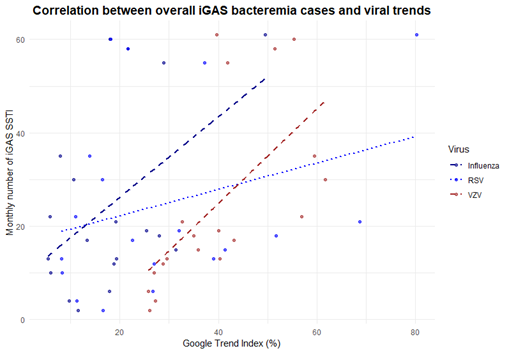

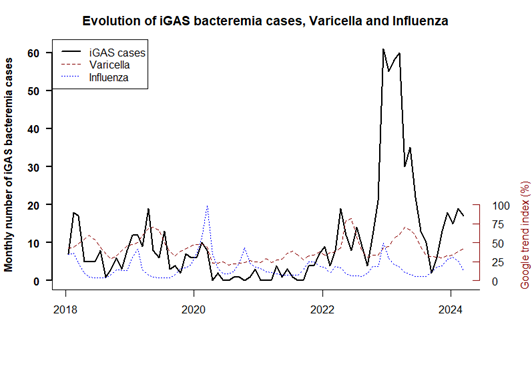


**e:** Graphical representation of the Pearson correlation between the monthly number of bacteriemia and sepsis iGAS cases and the relative trend of viruses (Influenza and VZV) during the post NPIs period.

**f:** Evolution of bacteriemia and sepsis iGAS cases (N=797) and VZV from January 1st, 2018, to March 31st, 2024

*Abbreviations: NPIs: non-pharmaceutical interventions; iGAS: invasive Group A streptococcus; ENT : Ear-nose-Throat*

# Appendix 17: Acknowledgement for participating study groups

The Dutch COPP-iGAS consortium consists of: E. van Kempen, M. van Veen (Haga Hospital, the Hague); A. Tulling, E. von Asmuth, J. van Prehn, E. Buddingh (Leiden University Medical Center, Leiden); L. van der Aa (Zaans Medical Center, Zaandam); E. Bijker (Maastricht University Medical Centre, Maastricht); Merijn Bijlsma, N. van Sorge (Amsterdam UMC, University of Amsterdam, Amsterdam); D. Borensztajn ( Northwest Clinics Alkmaar and Den Helder); C. Brackel (Tergooi Medical Center, Hilversum); B. de Gier (Centre for Infectious Disease Control, National Institute for Public Health and the Environment (RIVM), Bilthoven); M. van Houten, K. van de Weijer (Spaarne Gasthuis, Haarlem and Hoofddorp); M. Jacobs (Slingeland Ziekenhuis, Doetinchem); Marianne Koenraads (Haaglanden Medisch Centrum, the Hague); A. Kooter (Alrijne Ziekenhuis, Leiderdorp); A. Lebon (Albert Schweitzer Hospital, Dordrecht); J. van der Linden (Bernhoven, Uden); L. van Onzenoort-Bokken (Máxima Medisch Centrum Veldhoven); R. Oostenbrink, N. Boeddha (Erasmus MC Sophia, Rotterdam); K. Stol (Radboud University Medical Center, Nijmegen); G. Tramper-Stranders (Franciscus Gasthuis & Vlietland, Rotterdam); A. Verhage (University Medical Center Groningen, Groningen); J. Wildenbeest (University Medical Center Utrecht, Utrecht). We would like to thank the Netherlands Institute for Health Services Research (NIVEL), especially Marielle Hooiveld and the Dutch National Institute for Public Health and the Environment (RIVM), especially Saskia Bierman, for providing the Dutch national surveillance data. We would like to thank the Northwest Academy, Northwest Clinics Alkmaar and especially Wing Liu, for their invaluable support and guidance throughout this project.

The Spanish PEDGAS-NET working group consists of: Cristina Calvo, Isabel Mellado, David Grandioso (Hospital La Paz), Jesús Saavedra, David Aguilera, Elena Rincón, Ana Jové, Emilia Cercenado (Hospital General Universitario Gregorio Marañón), Francisco José Sanz Santaeufemia, María José Gónzalez, Elena Sánchez (Hospital Niño Jesús), Daniel Blázquez, Ángela Manzanares (Hospital 12 de Octubre), Rut Del Valle (Hospital Infanta Sofía), Elvira Cobo (Hospital Fundación de Alcorcón), Gloria Caro (Hospital Infanta Elena), Lucía Figueroa (Hospital General de Villalba), Marta Llorente (Hospital Universitario del Sureste), Pilar Galán (Hospital de Fuenlabrada), Arantxa Berzosa, Marta Illán (Hospital Clínico), Cristina Comín, Katie Badillo (Hospital de Torrejón), Fátima Ara Montojo (Hospital Puerta de Hierro), Ana Álvarez, Ignacio Callejas (Hospital de Getafe), Natalia Cerdeira (Hospital Universitario del Henares), Carmen Vázquez (Hospital Ramón y Cajal), Jaime Carrasco (Hospital Son Espases), Manuel Oltra, Anabel Piqueras (Hospital La Fe), Elena Montesinos (Consorcio Hospital General Universitario de Valencia), Nuria Benavente (Hospital Clínico Universitario de Valencia), César Gavilán (Hospital Universitario San Juan), Marta Dapena (Hospital de Castellón), Eloisa Cervantes, Ana Menasalvas, Genoveva Yagüe (Hospital La Arrixaca), Concha Rex (Hospital Universitario Santa Lucía), Victoria Rello, Mayli Lung (Hospital Vall D´Hebron), Anna Gamell (Hospital San Joan De Déu), Nuria López (Hospital del Mar), Borja Guarch, Anna Hernández (Hospital Josep Trueta), Berta Fernández, Carlos Rodrigo (Hospital Germans Trias i Pujol), Olga Calavia (Hospital Joan XXIII), Lola Falcón (Hospital Virgen del Rocío), Leticia Martínez (Complejo Hospitalario Torrecárdenas), Laura Martín, Begoña Carazo (Complejo Hospitalario Regional de Málaga), María Sánchez-Códez, Almudena Alonso (Hospital Universitario Puerta del Mar), Belén Sevilla, Federico García (Hospital San Cecilio), Beatriz Ruiz (Hospital Reina Sofía), Cristina Calvo Monge (Hospital Universitario Donostia de San Sebastián), María Itziar Pocheville (Hospital de Cruces), Laura Calle (Hospital Central de Asturias), Irene Rivero, Federico Martinón (Hospital Clínico Universitario de Santiago de Compostela) Elena Colino, Javier Cuenca (Complejo Hospitalario Materno-Insular), Marta Pareja (Hospital General de Albacete), Elena del Castillo (Hospital Materno Infantil de Badajoz), Beatriz Jiménez (Hospital Marqués de Valdecilla).

The Portuguese Study Group of Paediatric Invasive Streptococccal Disease consists of: Catarina Gouveia, Luis Varandas (Hospital Dona Estefania, Lisbon) and Ana Friães, Mário Ramirez (Faculdade de Medicina, Lisbon).
