# Supplementary material for: Paediatric invasive group A streptococcal infections and associations with viral infections in 15 European countries after lifting non-pharmaceutical interventions against SARS-CoV-2: an interrupted time-series analysis
Source: Lancet Reg Health Eur. 2025 Oct 18;59:101497. doi: 10.1016/j.lanepe.2025.101497 (PMC12569802; doi:10.1016/j.lanepe.2025.101497)
Supplement: Study Group Members List [file mmc2.docx]

**Additional authors of the PEGASUS study group**

| **First name** | **Last name** |
| --- | --- |
| Floor | Dekkers |
| Christian | Giske |
| Rachel | Hawkins |
| Olof | Hertting |
| Mojca | Kolnik |
| Enitan | Carrol |
| Nadia | Lewis-Burke |
| Pratham | Raghoenath |
| Samuel | Rhedin |
| Wouter | Rozemeijer |
| Katja | Seme |
| Natalia | Syrimi |
| Elizabeth-Barbara | Tatsi |
| Ivana | Velimirovic |
| Selma | Wilfing |
| Holger | Till |
| Mario | Ramirez |
| Ana | Friães |
