# Supplementary material for: Paediatric invasive group A streptococcal infections and associations with viral infections in 15 European countries after lifting non-pharmaceutical interventions against SARS-CoV-2: an interrupted time-series analysis
Source: Lancet Reg Health Eur. 2025 Oct 18;59:101497. doi: 10.1016/j.lanepe.2025.101497 (PMC12569802; doi:10.1016/j.lanepe.2025.101497)
Supplement: Pegasus Protocol [file mmc3.pdf]

Research protocol: PEGASUS: Paediatric European Group A Streptococcal United Study.

Version 1.4. 09-07-2024.

Principal investigator: Dorine Borensztajn, MD, MSc, PhD

PEGASUS: Paediatric European Group A Streptococcal United Study.

Sponsor: Noordwest Ziekenhuisgroep, Alkmaar, The Netherlands.

Co investigators

Austria

Benno Kohlmaier, MD, PhD, paediatrician. ORCID: 0000-0003-0685-689X

Volker Strenger, MD, paediatric infectious diseases specialist. ORCID: 0000-0002-6947-5894

Medical University of Graz, department of general paediatrics, Auenbruggerplatz 34/2, 8036, Graz.

Belgium

Anne Tilmanne

Denmark

Ulrikka Nygaard, MD, PhD, MPhil, paediatric infectious diseases specialist. ORCID: 0000-0002-2093-5909.

Copenhagen University Hospital, Rigshospitalet, department of paediatrics and adolescent medicine, Blegdamsvej 9, 2100, Copenhagen.

Mette Holm, MD, PhD, paediatric infectious diseases specialist. ORCID: 0000-0002-9159-4056.

Aarhus University Hospital, department of paediatrics and adolescent medicine,

Palle Juul-Jensens Boulevard 99, 8200, Aarhus.

France

Léa Lenglart, MD, paediatric emergency physician. ORCID: 0000-0003-2325-8998

Hôpital Universitaire Robert Debré, Paediatric Emergency Department, 48 Boulevard Sérurier, 75019, Paris.

Prof. Naïm Ouldali, MD, PhD, Pediatric Infectious Diseases specialist. ORCID: 0000-0003-4996-9640

Hôpital Universitaire Robert Debré, Department of general paediatrics and paediatric infectious diseases, 48 Boulevard Sérurier, 75019, Paris.

Yannis Lassoued

Germany

Ulrich von Both, MD, FRCPCH, paediatric infectious diseases specialist. ORCID: 0000-0001-8411-1071. LMU University Hospital Munich, department of paediatric infectious diseases, Lindwurmstraße 4, 80337, München.

Greece

Prof. Maria Tsolia, MD, PhD, paediatric infectious diseases specialist. ORCID: 0000-0003-2485-4409.
Angeliki (Kelly) Syngelou, MD, PhD, paediatric infectious diseases specialist.
Natalia Syrimi, MD, paediatric infectious diseases specialist. ORCID: 0000-0001-7011-5303.
P. and A. Kyriakou Children's Hospital, National and Kapodistrian University of Athens (NKUA), Second Dept of Paediatrics, Thivon and Levadias St, Goudi, 11527, Athens.

Irini Eleftheriou, MD, paediatrician. ORCID: 0000-0002-5093-8502.
Mitera Hospital, department of pediatrics, Erithrou Stavrou, 15123, Athens.

Prof. Nikos Spyridis MD, PhD, paediatric infectious diseases specialist. ORCID: 0000-0002-1403-5699.
Mitera Hospital, department of pediatrics, Erithrou Stavrou, 15123, Athens.

Prof. Athanasios Michos, MD, PhD, paediatric infectious diseases specialist. ORCID: 0000-0003-1745-1118.
Aghia Sophia Children's Hospital, National and Kapodistrian University of Athens, first department of paediatrics, Thivon and Levadeias, 11526, Athens.

Patra Koletsi, MD, MPH. Paediatric infectious diseases specialist. ORCID: 0000-0003-1012-2290.
Penteli Children's Hospital, PICU, 8 Hippokrates str, 15236, Athens.

Iceland

Valtýr Stefánsson Thors, MD, PhD, Paediatric infectious diseases specialist, ORCID 0000-0002-0976-9604. Children's hospital Iceland, department of Infectious Diseases, Hringbaut, 101, Reykjavik.

Ireland

Cilian O Maoldomhnaigh

Italy

Silvia Bressan, MD, PhD, paediatric emergency physician. ORCID: 0000-0002-6736-5392.
University of Padova Hospital, department of paediatric emergency medicine, via Giustiniani 3, 35128, Padova.

Daniele Donà, MD, PhD, MSc, paediatric infectious diseases specialist. ORCID: 0000-0001-7105-2105.
University Hospital of Padua, department for Women's and Children's Health, University Hospital of Padua, Via Giustiniani 2, 35128, Padua.

Danilo Buonsenso, MD, PhD, paediatric infectious diseases specialist. ORCID: 0000-0001-8567-2639.

Fondazione Policlinico Universitario A. Gemelli IRCCS, department of Woman and Child Health and Public Health, Largo A. Gemelli 8, 00168, Rome.

Riccardo Castagnoli

Latvia

Prof. Dace Zavadska, MD, PhD, paediatric infectious diseases specialist. ORCID: 0000-0003-4892-3763.
Riga Stradins university; Children University Hospital Riga, department of Paediatrics, Vienibas gatve 45, LV1004, Riga.

Anda Nagle, MD, paediatric infectious diseases specialist.
Children University Hospital Riga , department of paediatric and infectious diseases, Vienības gatve 45, LV1004, Riga.

Marta Daina, researcher. ORCID: 0009-0008-6637-3658. Riga Stradins university, faculty of medicine, Vienības gatve 45, LV1004, Riga.

The Netherlands

COPP-iGAS network

Dorine Borensztajn, MD, PhD, MSc, paediatric emergency physician. ORCID: 0000-0002-2437-0757.
Erasmus Medical Centre Sophia Children's Hospital, department of general paediatrics, Dr. Molewaterplein 60, 3015 GJ Rotterdam.
Noordwest Ziekenhuisgroep, department of paediatrics, Wilhelminalaan 12 1815 JD Alkmaar.

Maasstad Ziekenhuis, department of paediatrics, Maasstadweg 21 3079 DZ Rotterdam.

Jeroen Hol, MD, PhD, paediatric emergency physician. ORCID: 0000-0003-4481-7250.
Floor Dekkers, medical student.
Izel Özmen, researcher.
Noordwest Ziekenhuisgroep, department of paediatrics, Wilhelminalaan 12 1815 JD Alkmaar.

Wouter Rozemeijer, MD, clinical microbiologist.
Noordwest Ziekenhuisgroep, department of medical microbiology, Wilhelminalaan 12 1815 JD Alkmaar.

Navin Boeddha, MD, PhD, paediatrician. ORCID: 0000-0001-9487-1218.
Maasstad Hospital, department of paediatrics, Maasstadweg 21, 3079 DZ, Rotterdam.
Erasmus Medical Centre Sophia Children's Hospital, department of general paediatrics, Dr. Molewaterplein 40, 3015 GD Rotterdam.

Rianne Oostenbrink, MD, PhD, paediatrician. ORCID: 0000000179198934
Erasmus Medical Centre Sophia Children's Hospital, department of general paediatrics, Dr. Molewaterplein 40, 3015 GD Rotterdam.

Evelien van Kempen, MD, PhD student, paediatric resident. ORCID: 0000-0002-7416-795X.
Mirjam van Veen, MD, PhD, paediatrician: ORCID: 0009-0004-1084-3447.
Juliana Children's Hospital Haga Hospital, department of paediatrics, Els Borst Eilersplein 275, 2545 AA, the Hague, the Netherlands

Emmeline Buddingh, MD, PhD, paediatric infectious diseases specialist. ORCID: 0000-0002-2755-9408
LUMC,department of paediatric infectious diseases, Albinusdreef 2, 2333 ZA Leiden.

Poland

Kamila Ludwikowska, MD, PhD. ORCID: 0000-0002-4128-3401.

Monika Tokarczyk, MD.

Wroclaw Medical University, department of paediatric Infectious Diseases, Chalubinskiego 2-2a, 50-368, Wroclaw, Poland.

Portugal

Catharina Gouveia, MD, PhD, paediatric infectious diseases specialist. ORCID: 0000-0002-4162-8473.

Hospital Dona Estefania, department of paediatrics, Rua Jacinta Marto, 1169-045, Lisbon.

Slovenia

Marko Pokorn, MD, PhD, paediatric infectious diseases specialist. ORCID: 0000-0002-2341-2791.

Mojca Kolnik, MD. Researcher. ORCID: 0000-0003-4194-7295.

University Medical Center Ljubljana, Division of Paediatrics, Bohoričeva 20, 1000, Ljubljana.

Katarina Vincek, MD, paediatrician. ORCID: 0000-0003-2146-1829.

University medical center Ljubljana, Slovenia, department of infectious diseases, Japljeva ulica 2, 1000, Ljubljana.

Tina Plankar Srovin, MD, PhD, paediatrician. ORCID: 0000-0001-7911-696X.

University Medical Centre Ljubljana, department of infectious diseases, Japljeva 2, 1241, Ljubljana.

Spain

PedGAS network

Irene Rivero Calle, MD, PhD, paediatric infectious diseases specialist. ORCID: 0000-0002-3678-9264.

Hospital Clínico Universitario de Santiago de Compostela, Translational Paediatrics and Infectious Diseases Section, Travesia de Choupana S/N, 15706, Santiago de Compostela.

Prof. Federico Martinón-Torres, paediatrian, MD, PhD. ORCID: 0000-0002-9023-581X.

Hospital Clínico Universitario de Santiago, department of Paediatrics, Choupana s.n., 15701, Santiago de Compostela.

Ana Isabel Dacosta Urbieta, MD, paediatric infectious diseases specialist. ORCID: 0000-0002-8803-957X.

Hospital Clinico de Santiago de Compostela, department of Paediatrics, Travesia da Choupana s/n, 15706, Santiago de Compostela.

Pablo Rojo, MD, PhD, paediatric infectious diseases specialist.

Daniel Blazquez Gamero, MD, PhD, paediatric infectious diseases specialist.

Adriana Shan, MD, paediatric infectious diseases specialist.

Hospital Universitario 12 de Octubre, department of paediatric infectious diseases, Av. de Córdoba, s/n, 28041, Madrid

Cristina Calvo, MD, PhD, paediatric infectious diseases specialist. ORCID: 0000-0002-6503-3423.
Hospital Universitario La Paz, department of paediatric infectious diseases, Pº Castellana, 261, 28046 Madrid

Jesus Saavedra, MD, PhD, paediatric infectious diseases specialist. ORCID: 0000-0001-5971-011X

David Aguilera-Alonso, MD, PhD, paediatric infectious diseases specialist. ORCID: 0000-0003-1017-2386
Gregorio Marañón Hospital, department of paediatrics, O'Donell 48-50, 28009, Madrid, Spain

Sweden

Samuel Rhedin, MD, PhD, paediatrician. ORCID: 0000-0001-5798-2875

Sachs' Children and Youth Hospital, Nobels väg 12A, 171 65, Stockholm.

Karolinska Institutet, department of medical epidemiology and biostatistics, Stockholm.

Prof. Christian Giske, MD, PhD, clinical microbiologist. ORCID: 0000-0003-4327-6122

Karolinska Institutet and Karolinska University Hospital, Alfred Nobels Allé 8, 14186, Stockholm.

Olof Hertting, MD, PhD, paediatric infectious diseases specialist. ORCID: 0000-0002-0813-7998.

Karolinska University Hospital, department of paediatric infectious diseases, Eugeniavägen 23 17169, Stockholm.

Switzerland

Anita Niederer-Loher, MD, paediatric infectious diseases specialist. ORCID: 0000-0002-5985-5682

Christian R. Kahlert, MD, PD, paediatric infectious diseases specialist. ORCID: 0000-0002-0784-3276

Children's Hospital of Eastern Switzerland, St. Gallen, division of paediatric infectious diseases and hospital epidemiology, Claudiusstrasse 6, 9006, St. Gallen
Swiss Pediatric surveillance unit (SPSU).

UK

Ruud Nijman, MD, PhD, MSc. Paediatric Emergency Physician. ORCID: 0000-0001-9671-8161

Research protocol: PEGASUS: Paediatric European Group A Streptococcal United Study.

Version 1.4. 09-07-2024.

St Mary's hospital - Imperial College NHS Healthcare Trust, department of paediatric emergency medicine, Praed Street, W2 1NY, London.

Imperial College London, Faculty of Medicine, Department of Infectious Diseases, Section of Paediatric Infectious Diseases, London.

Imperial College, Centre for Paediatrics and Child Health, London.

Mohammed Zaman, MD. pediatric emergency physician. ORCID: 0000-0001-8626-9972
St. Mary's Hospital, Paediatric Emergency Medicine, Praed St, London, W2 1NY, London.

Prof. Enitan Carrol, MD. Paediatric Infectious Diseases specialist. ORCID: 0000-0001-8357-7726.

Alder Hey Children's Hospital, department of infectious diseases, East Prescot Road, L12 2AP, Liverpool.

Dr Andrew McArdle, MD, PhD. Paediatric Infectious Diseases specialist

Alder Hey Children's NHS Foundation Trust, department of Immunology and Infectious Diseases, Eaton Road, L12 2AP, Liverpool.

Nadia Lewis-Burke, BA (Hons), PGCert. ORCID: 0000-0002-6864-1918.

University of Liverpool, department of Infectious diseases, Eaton Road, L12 2AP, Liverpool.

Prof. Marieke Emonts, MD, PhD. Paediatric Infectious Diseases Specialist. ORCID: 0000-0002-2822-3527.

Newcastle upon Tyne NHS Hospitals Foundation Trust, Great North Children's Hospital, Paediatric Immunology, Infectious Diseases & Allergy; and Newcastle University,

Translational and Clinical Research Institute

Queen Victoria Road, RVI CRB level 4 block 2, NE1 4LP, Newcastle upon Tyne.

Christo Tsilifis, MD, MBBS. Paediatrician. ORCID 0000-0002-1368-104X.

Great North Children's Hospital. Paediatric Immunology & Infectious Diseases.

Royal Victoria Infirmary, Queen Victoria Road, NE1 4LP, Newcastle upon Tyne.

Carmen Ezinwoke, medical sudent. ORCID: 0009-0007-9540-1104

Newcastle University, Faculty of Medical Sciences,

Richardson Rd, NE2 4AZ, Newcastle upon Tyne

Abstract

Research protocol: PEGASUS: Paediatric European Group A Streptococcal United Study.

Version 1.4. 09-07-2024.

Introduction

Since 2022 there are reports of a rapid increase in invasive group A streptococcal infections (iGAS) in children in some but not all European countries. Detailed information on this increase is lacking. Furthermore, an increase in other invasive infections as well as the emergence of novel pathogens and disease entities is seen, such as MIS-C and severe hepatitis of unknown origin.

Aims

To set up a European research network and describe the incidence, risk factors, clinical phenotypes, microbiology and resistance, treatment, and outcomes of iGAS in children across Europe. The network will subsequently be utilized for early alerting regarding the rise of other invasive and emerging infections in children. It will enable swift data collection pertaining to patient characteristics, presentation, progression, and treatment.

Methods

International, retrospective cohort and observational surveillance study of children 0-18y attending the ED or admitted to the hospital with iGAS. Clinical and microbiological data, national vaccination schedules, and COVID non-pharmaceutical interventions (NPIs) will be collected and compared between countries. Subsequently, the network will be employed to raise alarms regarding the surge of other invasive infections or emerging infections in children. Anonymous data about these novel infections will be collected, similar to the data from iGAS (clinical characteristics and microbiological data). The study is a non-profit study.

Significance

Our study will describe variation in the clinical phenotypes of iGAS, will aid in the understanding of its association with COVID NPIs and allow for comparison between countries. Furthermore, it will aid in early recognition of invasive and emergent infectious diseases in children and the reduction of outbreaks.

Introduction

Since the COVID-19 pandemic and the implementation of non-pharmaceutical interventions (NPIs) to reduce the spread of SARS-CoV- 2, a secondary effect has also been reported on the epidemiology of other viruses and bacterial infections. [Kruizinga 2021; Cohen trends 2022] At first, a decrease was seen in paediatric infectious diseases, followed by an off-season rebound with increased frequency of several common paediatric infectious diseases, such as bronchiolitis and gastroenteritis. [Cohen trends 2022]

In line with this, in 2022 several European countries as well as the United States, reported a rapid increase in children presenting with invasive group A streptococcal infections (iGAS). [van Kempen 2022; WHO; UK surveillance; Ladhani 2022] Many of those children had severe disease needing paediatric intensive care unit (PICU) admission or causing mortality. Furthermore, it was noted that many of these children had a preceding infection with influenza virus or varicella zoster virus (VZV). (1)

One hypothesis explaining the increase in iGAS is related to the various NPIs introduced between 2020 and early 2022, with the aim of containing the COVID-19 epidemic, and consequently resulting in an 'immunity gap' in children. (2)

Although this increase was seen in several European countries [WHO; van Kempen 2022; UK Surveillance; Ladhani 2022] this has not been reported in all European countries. Possible explanations for differences between countries include differences in COVID NPIs, differences in vaccination schedules for VZV and influenza, differences in circulating M protein genes (*emm* types) or underreporting due to the lack of a systematic surveillance system.

Importantly, detailed and systematic data collection on the clinical phenotypes is lacking, which is essential to better understand the current incidence, risk factors and outcome across European. However, given the rapid increase of iGAS cases, the increased case fatality rate described in a recent study in the Netherlands [van Kempen 2022] and the fact that it is a treatable disease when recognized early, prompt collection and dissemination of data are imperative.

The study will provide in-depth data on the clinical phenotypes, their management and their outcomes, to support clinicians dealing with children with iGAS in their clinical decision making. Increased awareness and early recognition can be lifesaving for children with iGAS and real-time surveillance will help to reduce outbreaks. Furthermore, it will provide public health policy makers with information regarding future NPIs and optimal vaccination schedules regarding other viral infections that may precede iGAS.

iGAS disease is just one example of how the landscape of PID has changed in recent years, and it is currently in a state of ongoing evolution. Other examples include the emergence of novel pathogens and disease entities such as MIS-C and severe hepatitis of unknown origin.

We believe that our study will facilitate communication and data collection between countries and will facilitate responding promptly to international emergencies relating to rare childhood conditions where international studies can make a contribution to science or public health.
Furthermore, the European Centre for Disease Prevention and Control (ECDC) [ECDC] and the World Health Organization (WHO) [WHO] have recently urged for vigilance for a rise in iGAS cases.
Furthermore, our study will contribute to antimicrobial resistance data. Antimicrobial resistance is one of the top priorities of the European Union (EU).

Making use of existing and active research networks which will ensure commitment and success of the study.

Research questions

Primary research questions

1. What is the incidence of iGAS in children before, during and after COVID NPIs in different European countries?
2. Is there a change in clinical phenotypes and outcome during these periods?
3. Are there differences in clinical phenotypes and outcome between European countries?

Secondary research questions

4. Is there a change in *emm* types and other virulence factors during these periods?
5. Are there differences in antimicrobial resistance between periods and between countries?
6. Can we identify risk factors for iGAS infection (e.g., preceding infections, age)?
7. Can we explain differences between countries (e.g., differences in vaccination strategies, NPIs or *emm* types)?
8. Can we use this research network for early alarming on other invasive and emergent infectious diseases in children?

Plan of investigation

The study will consist of four parts.

1) Retrospective multicentre cohort study regarding the incidence, risk factors, clinical phenotypes, microbiology and resistance, treatment, and outcome of children with iGAS in different European countries
2) Observational surveillance study regarding the incidence, risk factors, clinical phenotypes, microbiology and resistance, treatment, and outcome of children with iGAS in different European countries
3) Setting survey, regarding national and regional aspects of healthcare possibly impacting iGAS incidence and the care for children with iGAS.
4) A surveillance study that registers the increase of other invasive or emergent infectious diseases.

The complete study is a non-profit study.

Retrospective study:
Participating centres will be asked to report all iGAS cases in the specified time period.
Anonymized data will be collected in an online database. Data will be combined into one dataset and then compared and analysed in combination and per country. Insights from the retrospective data collection phase will be used for the development and implementation of the surveillance part of the study.

Surveillance study:
An online database will be used that for all new iGAS cases. Data collection will take place retrospectively once a month. Reporting by the participating centres will take place once a month and reminders will be sent regularly.
The data will be published online on a monthly basis without information that can be traced back to individual patients. Online data will be displayed as numbers as well as visually.

Setting survey:
An online survey will be sent to the lead investigators of each participating centre to collect information on national and regional aspects of healthcare possibly impacting iGAS incidence and the care for children with iGAS, such as vaccination schedules and vaccination rates and primary care availability.

Surveillance study for other invasive or emergent infectious diseases
An online database will be used for other invasive or emergent infectious diseases. Data collection will take place retrospectively once a month. Reporting by the participating centres will take place once a month and reminders will be sent regularly.
The data will be published online on a monthly basis without information that can be traced back to individual patients. Online data will be displayed as numbers as well as visually.

Inclusion criteria:
- All children (1 month-18 years)
- attending the Emergency Department and/or admitted to the participating hospital - with iGAS infection or other invasive or emergent infectious disease.

Time period

1) January 2015 to April 2023*
2) April 2023 – onwards

* For the primary research questions, data collection will take place starting from 2018.
For the secondary research questions, data collection will take place from 2015 for those settings where this data is available and easily retrievable, for all other settings, data collection will take place from 2018.

Definitions

Confirmed iGAS infection:

- Clinical presentation consistent with iGAS* AND
- Isolation of group A streptococcus by culture or PCR or antigen detection test from a **normally sterile** body site

Probable iGAS infection:

- Clinical presentation consistent with STSS or necrotizing fasciitis AND
- Isolation of group A streptococcus by culture or PCR or antigen detection test from a **non-sterile body site** AND
- **No evidence of other pathogen** explaining the clinical presentation

***Clinical presentation consistent with iGAS:**
Clinically severe illness, **such as** sepsis, septic shock, STSS, pneumonia, meningitis, arthritis, osteomyelitis, myositis or necrotizing fasciitis

Other invasive infection:

- Clinical presentation consistent with severe disease (e.g. sepsis, meningitis, pneumonia) AND
- Isolation of a pathogen known to cause invasive disease by culture or PCR

Emergent infectious disease:

- Infectious diseases by previous unknown or undetected infectious agents that have recently appeared within a population or infectious diseases whose geographic range is rapidly increasing.

Index visit

The visit in which the sample is taken that later shows proof of group A streptococcus or another invasive/emergent pathogen.
This is not known at the time of visit, as these results take time.
This does not have to be the first visit in the disease episode.

Exception from informed consent

Given the following study characteristics, we would request an exception from informed consent.

- Data collection will take place retrospectively for the retrospective study as well as the surveillance study.
- Inclusion can only take place retrospectively, as upon patient presentation, culture results are not available yet.
- Data will be collected completely anonymously
- Data consist of routine care data
- No intervention will take place and collecting data will pose no risk to patients
- Asking parents informed consent whose child suffered from critical illness or has died will pose a considerable burden to them and can lead to response bias
- Data is intended for public health surveillance. The WHO states the following: “Individuals have an obligation to contribute to surveillance when reliable valid, complete data sets are required and relevant protection is in place. Under these circumstances consent is not ethically required.” (3)

Data collection and online database

The following variables will be collected: age, time of visit, gender, comorbidity, vaccination status, (viral) co-infection, e.g., VZV, influenza, respiratory syncytial virus (RSV), clinical symptoms, duration of symptoms before treatment, clinical diagnosis (e.g., pleural empyema, necrotising fasciitis, sepsis), culture/PCR/antigen detection results, *emm* type, antimicrobial resistance, treatment, hospital admission, ICU admission and death within 30 days.
During both the retrospective study as well as the surveillance study data will be collected anonymously in an online secure research database, hosted in the EU. Date of birth will be collected

as rounded age and time of visit will be collected as shift (day, evening, night), month and year of presentation to ensure patient data cannot be traced back to individual patients. To ensure data will remain truly anonymous, settings will be instructed to not store a local key that can link database data to local patient data.
In case of existing surveillance (UK, Switzerland), existing data will be exported anonymously and combined into a database containing data from all participating countries. Harmonization of data will take place to ensure the comparability and completeness of the collected data.
A survey per hospital will collect information on national and regional aspects of healthcare, such as vaccination schedules, vaccination rates, availability of primary care, funding of health care system, availability of rapid testing for group A streptococcal disease, and COVID NPI's.
After data collection, data will be stored and analyzed on a local secure hospital server of Noordwest Ziekenhuisgroep.

Statistical analysis

First, descriptive analyses will be performed. Changes over time will be assessed by interrupted time-series analysis models. [Lenglart 2022]
Associations between NPIs and changes over time in iGAS incidence will be assessed by seasonally adjusted multivariable quasi-Poisson regression modelling and will be displayed as incidence rate ratios (IRR). [Lenglart 2022]
We will apply regression analyses to identify risk factors for adverse outcome. Statistical programs (SPSS and R) will be used for statistical analysis.

Dissemination

Study results will be made available through scientific papers.
Furthermore, surveillance data will be made available online and updated regularly.
Online data will be displayed visually and an online dashboard will be used.
Dissemination will further be enhanced by the use of social media.

Timetable

Part I: Completion of research protocol	2023 Q1
Part I: Obtaining local medical ethical committee approval	2023 Q1
Part I: Collecting clinical information	2023 Q2

Part I: Analysing data	2023 Q3
Part I: Writing paper and dissemination of study results	2023 Q3-Q4
Part II: Developing international surveillance and dissemination system	2023 Q1-2
Part II: Implementing international surveillance and dissemination system	2023 Q3-4
Part II: Writing applications for additional funding	2023 Q1-4
Part III: Collecting setting data	2023 Q2
Part IV: Setting up a registry for other invasive and emergent infectious diseases	2023 Q4

Feasibility

- Previous successful collaboration with the same international research group has led to several publications. (1, 4–10)
- The Dutch study group on Invasive Group A streptococcal infections in children set up a national surveillance study on iGAS recently [van Kempen, 2022]. These experiences will be used to set up this international surveillance study.
- Previous collaborations with members of this research group have led to publications on the impact of NPIs on paediatric infectious diseases, including time-series analysis [Lenglart 2022]
- This collaboration includes researchers with different fields of expertise; paediatric infectious diseases specialists, paediatric emergency physicians, general paediatricians, microbiologists, specialists in public health/epidemiologists, IT specialists and statisticians.
- The applicant has received formal IT training and has successfully developed and implemented several IT tools for online international data collection. (6, 8)
- Given the limited number of required data per patient, the possibility to enter data online and the invasive nature of the disease we expect a high commitment and response rate

Significance of the study

Our study will describe changes in the clinical phenotypes of iGAS, will aid in the understanding of its association with COVID NPIs and allow for comparison between countries. It will identify risk factors for severe disease and will aid in early recognition of iGAS disease. Furthermore, it will aid in the early recognition of iGAS disease and the reduction of outbreaks. In addition to this, our study will contribute to antimicrobial resistance data. Antimicrobial resistance is one of the top priorities of the European Union (EU).

The COVID-19 pandemic seems to have had a profound impact on the epidemiology of paediatric infectious diseases in different ways, leading to several notable changes. (11)
Besides the increase in iGAS disease, an increase in other invasive pathogens, including meningococcal disease, is seen in the paediatric population. (11) These changes in the epidemiology of paediatric infectious diseases highlight the dynamic nature of microbial populations and the need for ongoing surveillance and adaptation in clinical practices and public health strategies.

Furthermore, seasonal shifts have been observed, of which the most notable one is the occurrence of unusual high respiratory syncytial virus (RSV) infections during the summer, which is typically considered an off-season for RSV. This atypical pattern of RSV infections has posed challenges in disease management and public health response. (11)

Additionally, the emergence of new clinical entities, such as Multisystem Inflammatory Syndrome in Children (MIS-C) (12–14) and severe acute hepatitis of unknown aetiology (15–17) has been observed. These novel conditions have presented clinicians with unique diagnostic and therapeutic challenges, further emphasizing the need for enhanced surveillance, research and collaboration of different countries and specialists with different backgrounds.

After setting up the framework for the collection of data on iGAS, the PEGASUS research network and framework that is initiated for iGAS disease, will be used to collect and share real time surveillance data on different invasive, novel and emergent paediatric infectious diseases across Europe.

References

1. Evelien B. van Kempen MD1 PCJB-VMDP, Dorine Borensztajn MD PhD3,4, Clementien L. Vermont MD PhD 5, Marjolijn S.W. Quaak MD5, Jo-Anne Janson MD6, Ianthe Maat MD7, Kim Stol MD PhD 8, Bart J.M. Vlaminckx MD PhD9, Jantien W. Wieringa MD10, Nina M. van Sorge Professor PharmD PhD11, 12, Navin P. Boeddha MD PhD 3, Mirjam van Veen MD PhD 1. Increase in invasive group a streptococcal infections in children in the netherlands, a survey among 7 hospitals in 2022. PIDJ. 2022;Accepted
2. European Paediatric Societies Call for an Implementation of Regular Vaccination Programs to Contrast the Immunity Debt Associated to Coronavirus Disease-2019 Pandemic in Children. [editorial]. J Pediatr 2022;242:260.
3. WHO. Increase in invasive Group A streptococcal infections among children in Europe, including fatalities. WHO. Available from: https://www.who.int/europe/news/item/12-12-2022-increase-in-invasive-group-a-streptococcal-infections-among-children-in-europe--including-fatalities
4. Rose K, Bressan S, Honeyford K et al. Responses of paediatricediatric emergency departments to the first wave of the COVID-19 pandemic in Europe: a cross-sectional survey study. BMJ Paediatr Open. 2021;5:e001269.
5. Borensztajn D, Hagedoorn NN, Carrol E et al. Characteristics and management of adolescents attending the ED with fever: a prospective multicentre study. BMJ Open. 2022;12:e053451.
6. Borensztajn D, Yeung S, Hagedoorn NN et al. Diversity in the emergency care for febrile children in Europe: a questionnaire study. BMJ Paediatr Open. 2019;3:e000456.
7. Hagedoorn NN, Borensztajn D, Nijman RG et al. Development and validation of a prediction model for invasive bacterial infections in febrile children at European Emergency Departments: MOFICHE, a prospective observational study. Arch Dis Child. 2021;106:641-647.
8. Borensztajn DM, Hagedoorn NN, Carrol ED et al. A NICE combination for predicting hospitalisation at the Emergency Department: a European multicentre observational study of febrile children. Lancet Reg Health Eur. 2021;8:100173.
9. Borensztajn DM, Hagedoorn NN, Carrol ED et al. Febrile children with comorbidities at the emergency department - a multicentre observational study. Eur J Pediatr. 2022;181:3491-3500.
10. Tan CD, Hagedoorn NN, Dewez JE et al. Rapid Viral Testing and Antibiotic Prescription in Febrile Children With Respiratory Symptoms Visiting Emergency Departments in Europe. Pediatr Infect Dis J. 2022;41:39-44.
11. Cohen PR, Rybak A, Werner A et al. Trends in paediatric ambulatory community acquired infections before and during COVID-19 pandemic: A prospective multicentric surveillance study in France. Lancet Reg Health Eur. 2022;22:100497.
12. Riphagen S, Gomez X, Gonzalez-Martinez C, Wilkinson N, Theocharis P. Hyperinflammatory shock in children during COVID-19 pandemic.[letter]. Lancet 2020;395(10237):1607-1608.
13. Kanthimathinathan HK, Scholefield BR. Paediatric Inflammatory Multisystem Syndrome: Time to Collaborate. J Paediatric Infect Dis Soc. 2021;10:227-229.
14. Verdoni L, Mazza A, Gervasoni A et al. An outbreak of severe Kawasaki-like disease at the Italian epicentre of the SARS-CoV-2 epidemic: an observational cohort study. Lancet. 2020;395:1771-1778.
15. Elsheikh R, Tien HT, Makram AM et al. Acute hepatitis of unknown origin in children: Behind the statistics. Hepatology. 2023;77:2118-2127.
16. Namakin K, Naserghandi A, Allameh SF. Severe acute hepatitis of unknown etiology in children in 2022: A Narrative Review. New Microbes New Infect. 2023;51:101087.

17. WHO. Available from: https://www.who.int/emergencies/disease-outbreak-news/item/2022-DON376
